# Delivering Progranulin to Astrocytic Lysosomes Promotes Growth of Co‐Cultured Neurons

**DOI:** 10.1111/jnc.70284

**Published:** 2025-11-03

**Authors:** Azariah K. Kaplelach, Justin A. Hall, Wren O. Nader, Amelia G. Davidson, Margaret D. Ireland, Lara Ianov, Andrew E. Arrant

**Affiliations:** ^1^ Killion Center for Neurodegeneration and Experimental Therapeutics, Alzheimer's Disease Center, Evelyn F. McKnight Brain Institute, Department of Neurology University of Alabama at Birmingham Birmingham Alabama USA; ^2^ Department of Neurobiology University of Alabama at Birmingham Birmingham Alabama USA

**Keywords:** astrocyte, frontotemporal dementia, lysosome, progranulin

## Abstract

Progranulin (*GRN*) mutations, most of which cause progranulin haploinsufficiency, are a major genetic cause of frontotemporal dementia (FTD). Restoring progranulin to people with *GRN* mutations is a promising therapeutic strategy and understanding progranulin's mechanism of action may enable the design of optimal progranulin‐based therapies. Progranulin is constitutively secreted and interacts with several receptors, but is also taken up and trafficked to lysosomes where it is necessary for maintaining normal lysosomal function. Progranulin promotes neuronal growth and survival, but it is not clear if these actions are mediated by extracellular signaling or by regulation of lysosomal function. In previous work we showed that progranulin acts in neuronal lysosomes to promote neuronal survival. In this study we investigated the mechanism by which progranulin promotes neuronal growth using lentiviral vectors expressing either progranulin (PGRN) or a non‐secreted, lysosome‐targeted progranulin (L‐PGRN) in rat primary hippocampal neurons and astrocytes. Using lentiviral vectors driven by non‐selective (PGK), neuron‐selective (hSyn), or astrocyte‐selective (GFAP) promoters, we found that delivering L‐PGRN to astrocytes, but not neurons, promoted dendritic outgrowth in primary hippocampal cultures. L‐PGRN–transduced astrocytes grown on transwell inserts also promoted the growth of co‐cultured neurons. RNA sequencing of astrocytes indicated that L‐PGRN downregulated transcriptomic pathways associated with cellular reactivity. Analysis of astrocyte conditioned medium showed that transduction with L‐PGRN reduced the secretion of PAI‐1, a protease inhibitor that inhibits neuronal outgrowth in hippocampal cultures. Collectively, these data indicate that delivering progranulin to astrocytic lysosomes may inhibit the secretion of factors that restrain neuronal outgrowth. Consistent with this hypothesis, depleting astrocytes from hippocampal cultures increased dendritic outgrowth and occluded the pro‐growth effects of L‐PGRN. These data show that under these culture conditions, progranulin secretion is not required to promote dendritic outgrowth. Instead, progranulin increased dendritic outgrowth by a non‐cell autonomous mechanism involving actions in astrocytic lysosomes. These data add to a growing body of evidence that progranulin may act on astrocytes to promote neuronal health.

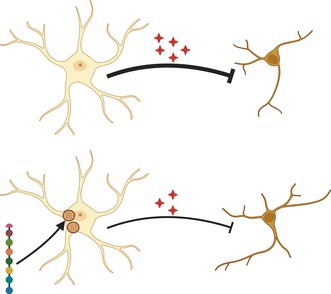

## Graphical abstract

Loss‐of‐function progranulin (*GRN*) mutations cause frontotemporal dementia. Most of these mutations cause haploinsufficiency of progranulin, a secreted pro‐protein that has neurotrophic and anti‐inflammatory effects. Progranulin is constitutively secreted before trafficking to lysosomes and it is unclear if its effects are mediated by extracellular signaling or by actions in lysosomes. Here, we show that progranulin enhances neuronal growth in primary hippocampal cultures via a non‐cell autonomous mechanism. Delivering progranulin to lysosomes of astrocytes, but not neurons, promoted dendritic growth of hippocampal neurons. Additional data indicated that these effects were mediated by reducing the secretion of astrocytic factors that restrain neuronal growth.

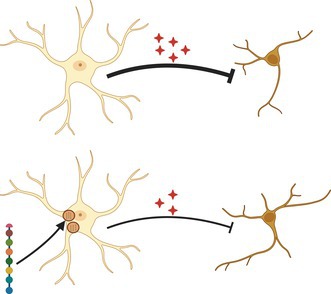

**Abbreviations**
Ara‐C: Cytosine β‐D‐arabinofuranoside
FTD: frontotemporal dementia
GFAP: glial fibrillary acidic protein
L‐PGRN: lysosome‐targeted progranulin
MAP 2: microtubule‐associated protein 2
PAI‐1: plasminogen activator inhibitor‐1
PGRN: progranulin
RRID: research resource identifier

## 1 Introduction

Loss‐of‐function mutations in progranulin (*GRN*) are a major genetic cause of frontotemporal dementia (FTD) (Baker et al. 2006; Cruts et al. 2006), a neurodegenerative disorder characterized by behavioral, cognitive, and/or language impairments (Gorno‐Tempini et al. 2011; Rascovsky et al. 2011). Most of these *GRN* mutations cause progranulin haploinsufficiency (Finch et al. 2009; Meeter et al. 2016). Progranulin‐insufficient mouse models develop FTD‐related pathology and behavioral deficits, implicating progranulin haploinsufficiency as the mechanism by which *GRN* mutations cause FTD (Ahmed et al. 2010; Filiano et al. 2013; Ghoshal et al. 2012; Lui et al. 2016; Nguyen et al. 2018; Smith et al. 2024; Zhang et al. 2020). *GRN* polymorphisms are associated with increased risk for Alzheimer's disease (Bellenguez et al. 2022) and Parkinson's disease (Nalls et al. 2019), and may also influence cognitive aging (Rhinn and Abeliovich 2017; Tesi et al. 2024). A common *GRN* polymorphism associated with risk for AD and FTD modestly reduces progranulin levels (Nicholson et al. 2014; Rademakers et al. 2008), indicating that even mild reduction of progranulin may increase risk for neurodegenerative disease. Collectively, these data indicate that progranulin exerts important protective effects in the aging brain.

Progranulin exerts anti‐inflammatory (Martens et al. 2012; Yin et al. 2010) and neurotrophic (Gass et al. 2012; Van Damme et al. 2008) effects that may be protective in the aging brain. Loss of these effects may explain the relationship of low progranulin levels and neurodegenerative disease. Progranulin‐boosting therapies are therefore in development to restore progranulin to people with *GRN* mutations and have generated promising results in preclinical studies (Arrant et al. 2017, 2018; Kurnellas et al. 2023; Logan et al. 2021; Reich et al. 2024; Rosenthal et al. 2024; Sevigny et al. 2024; Tesla et al. 2024). Understanding the mechanism of progranulin's protective effects in the brain could enable optimization of these promising therapies.

Progranulin's protective effects may be mediated by several mechanisms. Progranulin localizes to lysosomes and is necessary for maintaining lysosomal function, as people with mutations on both *GRN* alleles develop a lysosomal storage disorder, neuronal ceroid lipofuscinosis (Huin et al. 2019; Smith et al. 2012). Progranulin stimulates the activity of multiple lysosomal enzymes, including the protease cathepsin D and several sphingolipid‐metabolizing enzymes (Arrant et al. 2019; Beel et al. 2017; Boland et al. 2022; Jian et al. 2016; Logan et al. 2021; Valdez et al. 2017, 2020; Zhou et al. 2017, 2019). However, progranulin is constitutively secreted before trafficking to lysosomes, and may interact with several signaling receptors (Altmann, Vasic, et al. 2016; Chen et al. 2013; Etemadi et al. 2013; Neill et al. 2016; Tang et al. 2011).

There is evidence that progranulin may act either in lysosomes or through extracellular signaling to restrain inflammation and promote neuronal growth and survival. Microglia and macrophages from *Grn* ^*−/−*^ mice have a reactive phenotype that may be driven by lysosomal dysregulation (Gotzl et al. 2018; Lui et al. 2016; Tanaka et al. 2014; Wu et al. 2021; Zhang et al. 2020). *Grn* ^*−/−*^ neurons have impaired autophagy/lysosomal function that may increase their susceptibility to injury (Altmann, Hardt, et al. 2016; Beel et al. 2017). However, delivering excess progranulin to wild‐type neurons enhances neuronal growth, promotes recovery from injury, and protects against neuronal death (Altmann, Hardt, et al. 2016; Altmann, Vasic, et al. 2016; Gao et al. 2010; Gass et al. 2012; Guo et al. 2010; Van Damme et al. 2008; Xu et al. 2011), perhaps by activating pro‐survival signaling cascades via extracellular signaling (Altmann, Vasic, et al. 2016; Gao et al. 2010; Xu et al. 2011).

Using a molecular targeting strategy based on the lysosomal membrane protein LAMP‐1 (Mikulka et al. 2020), we previously generated lentiviral vectors expressing lysosome‐targeted progranulin (L‐PGRN) to investigate progranulin's mechanism of action (Davis et al. 2021). L‐PGRN comprises progranulin fused to the transmembrane domain and cytosolic tail of LAMP‐1, resulting in the delivery of progranulin to lysosomes without secretion. Using L‐PGRN in lentiviral vectors with either non‐specific or neuron‐specific promoters, we showed that progranulin acts in neuronal lysosomes to protect against excitotoxic cell death (Davis et al. 2021). In this study, we used a similar strategy to investigate the mechanism underlying another well‐documented neurotrophic effect of progranulin, stimulation of neuronal growth (Gao et al. 2010; Gass et al. 2012; Hyung et al. 2019; Van Damme et al. 2008).

## 2 Methods

### 2.1 Animals

Pregnant female Sprague–Dawley rats (E16–17 upon arrival) were obtained from Charles River Laboratories (strain code #001). Rats were housed for 2–3 days with free access to food (Envigo Cat. #7917) and water in a facility accredited by the Association for Assessment and Accreditation of Laboratory Animal Care International before euthanizing to collect embryos for primary cultures at E19. Rats were euthanized by anesthetizing with isoflurane (5% by inhalation), followed by decapitation. Embryos were quickly removed and immediately decapitated to obtain brains for primary culture as described below. This procedure was approved by the University of Alabama at Birmingham Institutional Animal Care and Use Committee (Protocol #22553).

### 2.2 Primary Hippocampal Cultures

Each independent culture was obtained from a single rat, with all embryos from that rat pooled to obtain a single batch of cells. Thus, each independent culture reported in the figure legends represents one dam. The number of embryos varied per dam, but typically ranged from 6 to 12 embryos per dam to obtain a single culture. Brains were removed from E19 embryos and placed in ice‐cold Hibernate E medium (Thermo Fisher, Cat. # A1247601). Hippocampi were dissected and digested with 20 units of papain (Worthington Biochemical Corporation, Cat. # LK003176) before dissociation by triturating with a 1 mL pipette tip. Cells were then diluted with Trypan Blue (Thermo Fisher), counted on a hemocytometer, and seeded onto 12 mm No. 1.5 glass coverslips at a density of 10 000 cells/coverslip. For confirmation of progranulin expression by immunoblot, cells were seeded into 24‐well plates (Corning) at a density of 250 000 cells/well. Both coverslips and plates were coated with poly‐D‐lysine (MilliporeSigma Cat. #P6407) before use at 0.67 mg/mL for coverslips and 0.1 mg/mL for plates.

Cells were initially plated in Neurobasal A medium (Thermo Fisher, Cat. # 10888022) with B27 supplement (Thermo Fisher, Cat. # 17504044), 2 mM L‐glutamine (Thermo Fisher), and 10% fetal bovine serum (FBS, Biotechne, Cat. #S11550). After 2 h, cultures were subjected to two 50% media changes using the same medium without FBS, resulting in a final serum concentration of approximately 2.5%. Preliminary experiments with recombinant progranulin (R&D Systems Cat. #2420‐PG) incorporated recombinant progranulin into this media change at 10 ng/mL, and in a second 50% media change on DIV2. Lentiviral transduction was also incorporated into the initial media change as previously described (Davis et al. 2021). Treatments were assigned to wells in a recurring pattern such that treatment groups were evenly distributed across the rows and columns of the plates. Cultures were maintained at 37°C with 5% CO_2_.

### 2.3 Primary Astrocyte Cultures

Primary astrocytes were cultured using a method designed to produce in vivo‐like astrocytes (Wolfes et al. 2017; Wolfes and Dean 2018). Hippocampi from E19 embryos were isolated as described above, then digested with 0.1% trypsin (Thermo Fisher) and 0.5 mg/mL DNase I (MilliporeSigma) and triturated with a fire‐polished glass pipette. The resulting cells were filtered through a 70 μm cell strainer then cultured in T75 flasks (Corning) in DMEM (Corning) with 10% FBS (Biotechne) and 1% penicillin/streptomycin (Thermo Fisher). After 7 days, flasks were placed on a 240 rpm orbital shaker at 37°C for 6 h, washed with PBS, and passaged to either transwell inserts, 24‐well plates, or 12 mm glass coverslips that were pre‐coated with poly‐D‐lysine as described above. Astrocytes were seeded in all conditions at 5000 cells/well, and were cultured in Neurobasal medium (Thermo Fisher) with B27 supplement (Thermo Fisher), 2 mM L‐glutamine (Thermo Fisher), 1% penicillin/streptomycin (Thermo Fisher), and 5 ng/mL heparin‐binding EGF‐like growth factor (HBEGF, MilliporeSigma, Cat. #E4643). Lentiviral transduction was performed as described above.

### 2.4 Lentiviral Vectors

Generation of the PGK (RRIDs: Addgene_234872 and Addgene_234873) and hSyn (RRIDs: Addgene_234875, Addgene_234876, and Addgene_234877) lentiviral vectors used in this study is described in a previous publication (Davis et al. 2021). The PGK lentiviral vectors were derived from pRRLSIN.cPPT.PGK‐GFP.WPRE (RRID: Addgene_12252, a gift from Didier Trono), which served as the control GFP vector for these studies. GFAP lentiviral vectors were generated by removing the hSyn promoter from our hSyn lentiviral vectors by digestion with PacI and XhoI (New England Biolabs), and replacing it with the GFAP promoter. The GFAP promoter was amplified from the vector pAAV‐GFAP‐EGFP (RRID: Addgene_50473, a gift from Bryan Roth) using the following primers: for generating GFAP‐IRES‐GFP, F:GATCCAGTTTGGTTAGATCTAACATATCCTGGTGTGGAG, R:GAAGCTTGAGCTCGACCCCGCGAGCAGCGGAGG; for generating GFAP‐PGRN‐IRES‐GFP and GFAP‐L‐PGRN‐IRES‐GFP, F:GATCCAGTTTGGTTAGATCTAACATATCCTGGTGTGGAG, R:CACATGGTGGCTCGACCCCGCGAGCAGCGGAGG. The resulting amplicons were cloned into the cut hSyn vectors using In‐Fusion HD (Takara Bio, Cat. #639650). The GFAP‐LGFP vector was generated by restriction digest of the control GFAP vector with BstBI and BamHI (New England Biolabs) and insertion of an L‐GFP amplicon generated from a PGK L‐GFP vector (RRID:Addgene_234874) with the following primers: F:CGAGCTCAAGCTTCGGCCACCATGGTGAGCAAGG, R: GGAGAGGGGCGGATCCTACTAGATAGTCTGGTAGCCTGCG using In‐Fusion HD (Takara Bio).

All lentiviral vectors were packaged as previously described (Davis et al. 2021) by co‐transfecting HEK 293T cells (ATCC #CRL‐3216) with the desired transfer vector and the packaging vectors pMD2.G (RRID:Addgene_12259, a gift from Didier Trono) and psPAX2 (RRID:Addgene_12260, a gift from Didier Trono). Lentiviral vectors were concentrated by ultracentrifugation before titers were determined by qPCR using the Lenti‐X qRT‐PCR titration kit (Takara Bio, Cat. #639650).

### 2.5 Immunostaining

Cultures grown on glass coverslips were fixed in 4% paraformaldehyde (MilliporeSigma) with 4% sucrose (Fisher Scientific) for 30 min at room temperature before immunostaining. After blocking for 1 h, the coverslips were incubated overnight with primary antibody. The following primary antibodies were used for immunostaining: MAP2 (Thermo Fisher # PA1‐10005, RRID:AB_1076848), progranulin (R&D systems #AF2420, RRID:AB_2114489), GFAP (Agilent # Z0334, RRID:AB_10013382), S100β (Abcam #ab52642, RRID:AB_882426), HA tag (Cell Signaling Technologies #3724, RRID:AB_1549585), and Cathepsin D (R&D Systems #AF1029, RRID:AB_2087094). Blocking/antibody solutions were 5% normal goat serum, 5% donor horse serum, 1% bovine serum albumin, and 0.5% saponin for MAP2, and 3% bovine serum albumin with 0.5% saponin for all other antibodies. The following day, coverslips were incubated with species‐matched secondary antibodies conjugated to AlexaFluor 488 or 594 (Thermo Fisher or Jackson Immunoresearch), then mounted onto slides using Prolong Gold mounting medium with DAPI (Thermo Fisher).

### 2.6 Analysis of Neuronal Morphology

All neurons were imaged and analyzed by investigators blinded to the treatment group. Primary hippocampal cultures immunostained for MAP2 were imaged on an EVOS M5000 imaging system (Thermo Fisher). Four 20X z‐stacks were collected per coverslip. Maximum‐intensity projections of these z‐stacks were generated by EVOS imaging software and then analyzed with the SNT plug‐in on ImageJ (Arshadi et al. 2021). Neurons were manually traced using the following guidelines. First, the entire neuron was visible in the field of view and had no or minimal overlap with dendrites from other neurons. Second, the neuron appeared healthy and had no abnormalities such as blebbing or broken dendrites. Third, all neurons within each field of view that met these criteria were traced. Data were unblinded only after imaging and analysis were complete.

### 2.7 Immunoblotting

Cells were lysed with Ripa buffer and total protein content was determined by BCA Assay (Thermo Fisher) before diluting to a uniform protein concentration in Laemmli buffer. Samples were then loaded onto 10% tris‐glycine gels (Bio‐Rad) for SDS‐PAGE prior to transferring to low‐fluorescence PVDF membranes (Thermo Fisher). After blocking with protein‐free blocking buffer (Thermo Fisher), blots were incubated overnight in primary antibody. Primary antibodies included progranulin (MilliporeSigma #HPA008763, RRID:AB_1850339), LC3 (Cell Signaling Technologies #12741, RRID: AB_2617131), and Gapdh (MilliporeSigma #MAB374, RRID:AB_2107445). The following day, blots were incubated in species‐matched secondary antibodies (Li‐Cor Biosciences) and scanned on an Odyssey scanner (Li‐Cor Biosciences). When necessary, blots were quantified using ImageStudio Lite software (Li‐Cor Biosciences).

### 2.8 Analysis of Cell Proliferation

Astrocytes were incubated for 24 h with 10 mM BrDU (Thermo Fisher), then fixed with 4% paraformaldehyde (MilliporeSigma) and 4% sucrose (Fisher Scientific) in PBS. The cells were permeabilized in PBS with 0.5% Triton X‐100 (Fisher Scientific) for 15 min, then subjected to successive 10 min incubations in 1N HCl (Fisher Scientific), 2N HCl, and citrate/phosphate buffer (pH 7.4) before immunostaining. Cells were immunostained for BrDU (Thermo Fisher # MA3‐071, RRID:AB_10986341) as described above, but with the following modifications. The blocking/antibody solution was 3% bovine serum albumin (Fisher Scientific) with 0.5% Triton X‐100, and cells were stained with 0.1 mg/mL DAPI to obtain strong nuclear labeling before mounting onto slides with Prolong Gold mounting medium without DAPI (Thermo Fisher).

### 2.9 β‐Hexosaminidase Activity

β‐hexosaminidase activity was determined as previously described (Arrant et al. 2019) using the fluorogenic substrate 4‐methylumbelliferyl‐2‐acetamido‐2‐deoxy‐β‐D‐glucopyranoside (MilliporeSigma, Cat. #69585). Cell lysates or conditioned media were incubated with substrate in sodium citrate buffer, pH 4.2 for 1 h at 37°C. Reactions were stopped by addition of 0.2 M glycine, 0.2 M sodium carbonate (Wendeler and Sandhoff 2009). Fluorescence was read at 360 nm excitation/440 nm emission using a Biotek Synergy LX plate reader, and activity was quantified using a standard curve of 4‐methylumbelliferone run on each plate.

### 2.10 Cytokine Array

Conditioned media from astrocyte/neuron co‐cultures was analyzed using Proteome Profiler Rat XL Cytokine Arrays (R&D Systems, Cat. #ARY030) according to the manufacturer's instructions. Chemiluminescent signal was imaged on a Bio‐Rad Chemi‐Doc imaging system, and optical density of array signal was quantified with ImageJ software (Schindelin et al. 2012).

### 2.11 ELISA

Astrocyte conditioned media was analyzed using a Rat PAI‐1 ELISA kit (Abcam Cat. # ab201283) and a Human Progranulin Duoset ELISA kit (R&D Systems Cat. #DY2420) according to the manufacturers' instructions. Human progranulin ELISA plates were developed using 1‐step Ultra TMB‐ELISA substrate (ThermoFisher). ELISA plates were read at 450 nm on a Biotek Synergy LX plate reader and quantified based on standard curves run on each plate using Biotek Gen5 software.

### 2.12 RNA Sequencing

Bulk RNA sequencing was conducted on four independent cultures of primary astrocytes, with paired samples from each culture treated with lenti‐PGK‐GFP or lenti‐PGK‐L‐PGRN. RNA was isolated from each culture using a Qiagen RNeasy mini kit, then sequenced at the UAB Heflin Center for Genomic Sciences Core Laboratory. mRNA was processed for poly‐A selection before libraries were prepared using the NEB Next Ultra II Directional kit (New England Biolabs), according to the manufacturer's instructions. Samples were then analyzed by paired‐end sequencing using an Illumina NovaSeq 6000 (~46.5 M reads/sample).

### 2.13 Analysis of RNA Sequencing

FASTQ files were analyzed at UAB's Biological Data Science Core (RRID:SCR_021766) using the nf‐core/rnaseq pipeline, v.3.1.0 (Ewels et al. 2020). After assessing read quality, trimming, and read contamination removal with BBSplit (v 39.01), files were aligned to the rn7 genome (v107) using STAR (v 2.7.9a) and the reads were quantified with Salmon (v 1.9.0) with the following parameters enabled: ‘‐‐seqBias ‐‐gcBias’. Downstream analysis was performed in R (v 4.3.0) in a Singularity container. Specifically, the quantified counts were imported with tximport (v 1.28.0) and differential gene expression was assessed using limma‐voom (v 3.56.2). Criteria for differential expression were a log2 fold change of ±0.5 relative to control and an FDR‐adjusted *p* value < 0.05.

Gene set enrichment analysis (GSEA) (Subramanian et al. 2005) was conducted on all detected transcripts, ranked by fold change, using terms from the mouse molecular signatures (Liberzon et al. 2015) database. GSEA was conducted using clusterProfiler (Yu et al. 2012), with *p* values adjusted for false discovery rate by the Benjamini‐Hochberg method. The criterion for statistical significance was an FDR‐corrected *p* value < 0.05.

We also qualitatively assessed counts of cell‐type–specific genes from primary astrocyte cultures as an indicator of potential contamination by other cell types. We compared counts of the following well‐validated cellular markers: astrocytes (*Aldoc*, *Aqp4*, *Gfap*, *Gja1*, *S100b*, *Slc1a3*), microglia (*Aif1*, *C1qa*, *Cx3cr1*, *Itgam*, *Tmem119*), neurons (*Map2*, *Rbfox3*, *Syn1*, *Tubb3*), oligodendrocyte precursors (*Cspg4*, *Pdgfra*), oligodendrocytes (*Foxo4*, *Olig2*, *Plp1*).

### 2.14 Statistics

Prior to analysis, all data were tested for normality using the Shapiro–Wilk test and for equal variance using the Brown‐Forsythe test and Bartlett's test in GraphPad Prism 10. Data with non‐normal distribution and/or unequal variance between groups were log transformed before further analysis. Due to the inherent variability of neuronal growth within cultures, no outlier tests were performed and no neurons were excluded from statistical analysis. All analyses calculated two‐tailed *p* values with significance set at *p* < 0.05.

For all experiments, independent cultures were defined as biological replicates. Most experiments included multiple observations per culture (either analysis of several technical replicates or tracing of many individual neurons), so data were blocked by culture for statistical analysis. MAP2+ dendritic length was analyzed by restricted maximum likelihood linear mixed effects model (REML), with lentivirus or progranulin dose as a fixed effect and culture as a random effect, using the lme4 package in R (Bates et al. 2015). For data with a significant main effect of lentivirus, pair‐wise comparison of treatment groups versus the control vector was performed using the lmertest package in R with single‐step correction for multiple comparisons (Kuznetsova et al. 2017). For Ara‐C–treated cultures, MAP2+ dendritic length and counts of MAP2+ and GFAP+ cells were analyzed by REML with lentivirus and Ara‐C as fixed effects and culture as a random effect. For data with significant main effects and interactions, pair‐wise comparisons were performed with Tukey's post hoc test using the multcomp package in R (Hothorn et al. 2008).

All graphs of MAP2+ dendritic length depict the total dendritic length per neuron. Violin/box plots show the distribution for all neurons analyzed under each condition. On box plots, the upper and lower borders represent the 75th and 25th percentiles of the distribution, respectively, and the line in the middle of the box represents the median. Dot plots represent the median dendritic length of each independent culture (Lord et al. 2020).

LC3‐II and β‐hexosaminidase activity were normalized to control vectors and analyzed by nested ANOVA or nested *t*‐test. Data from human progranulin and PAI‐1 ELISA were analyzed by ANOVA or paired *t*‐test of average values from each independent culture. Progranulin immunoblots and immunofluorescence in Ara‐C–treated cultures were analyzed by paired *t* test or repeated‐measures ANOVA of the average levels from each culture. Data with significant main effects were analyzed for pairwise comparisons using post hoc tests as described in the figure legends. ANOVA and *t* tests were conducted with GraphPad Prism 10.

## 3 Results

### 3.1 Replicating Progranulin's Pro‐Growth Effect

**FIGURE 1:**
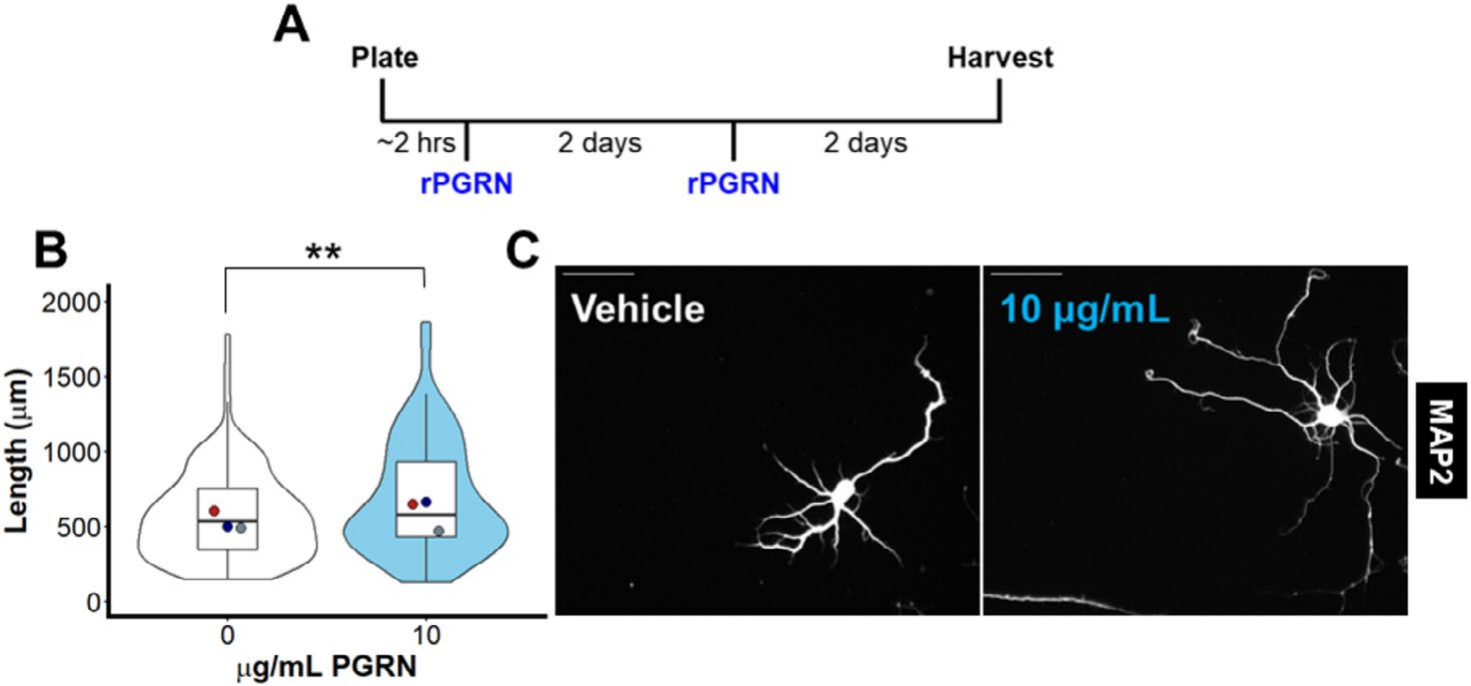
Recombinant progranulin stimulates dendritic outgrowth in primary hippocampal cultures. (A) Primary hippocampal cultures were plated onto coverslips, then subjected to media changes resulting in a final concentration of 0 or 10 μg/mL recombinant human progranulin. Cultures were maintained for a total of 4 days with progranulin levels replenished in a second media change to achieve final concentrations of 0 or 10 μg/mL recombinant human progranulin. (B) Neurons treated with recombinant progranulin had greater total dendritic length after 4 days in culture than neurons not treated with recombinant progranulin (linear mixed effects model, *t*_(217)_ = 2.619, *p* = 0.00945, *n* = 105–116 neurons per group from three independent cultures). Violin and box plots represent the distribution of total dendritic length for all neurons analyzed, and dots represent the median length of all neurons from each culture. Representative images of MAP2 immunostaining are shown in (C) with 50 μm scale bars.

Progranulin stimulates growth of cultured neurons under a variety of conditions (De Muynck et al. 2013; Gao et al. 2010; Gass et al. 2012; Hyung et al. 2019; Van Damme et al. 2008), so we initially tested culture conditions and analysis protocols that would allow us to detect this effect. We found that maintaining low‐density primary hippocampal cultures for 4 days, with the addition of recombinant progranulin to culture media after plating and on Day 2, resulted in increased total dendritic outgrowth of progranulin‐treated neurons versus vehicle‐treated controls (Figure 1). Despite the increase in total dendritic length, we did not observe significant changes in either dendritic branching (Figure S1a) or the number of primary dendrites (Figure S1b).

### 3.2 Delivering Progranulin to Lysosomes Promotes Dendritic Outgrowth

**FIGURE 2:**
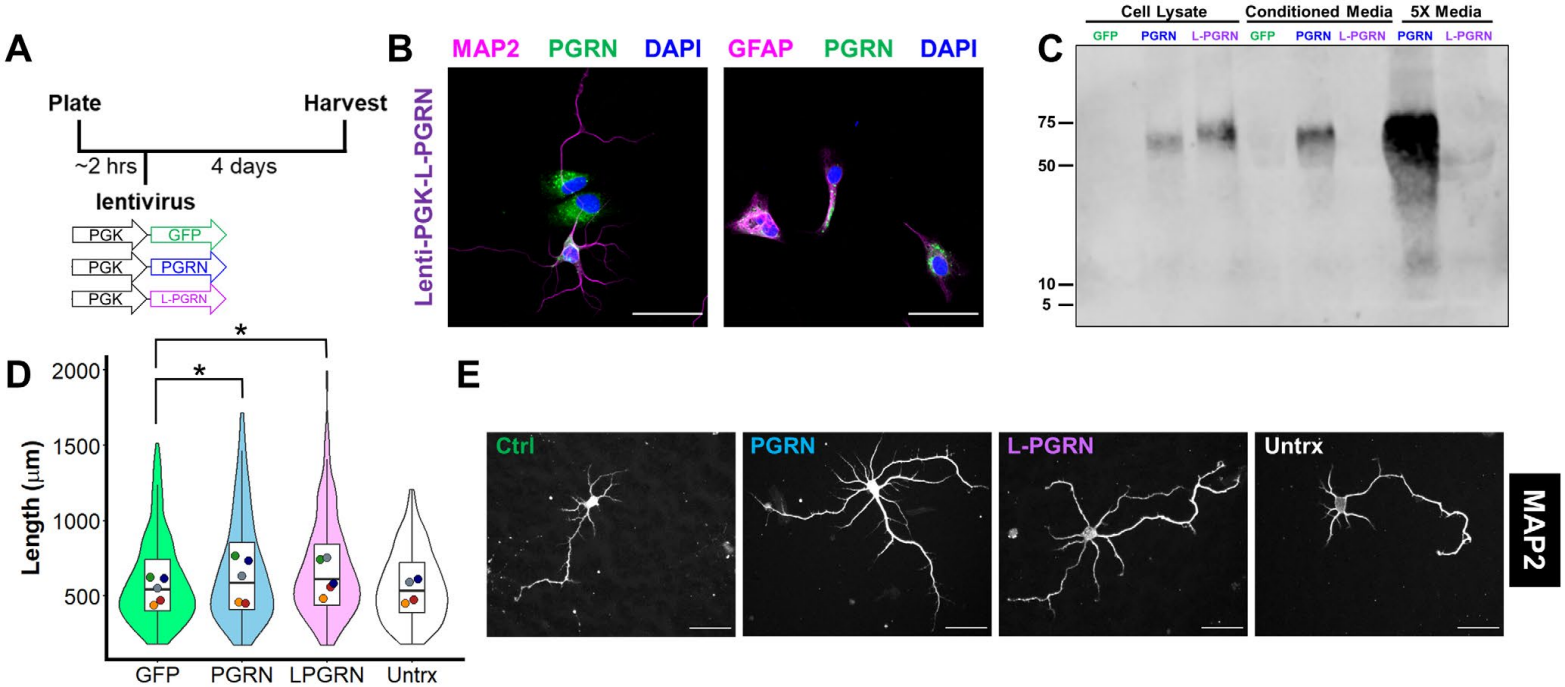
Delivering progranulin to lysosomes stimulates dendritic outgrowth. (A) Primary hippocampal cultures were plated onto coverslips, then transduced with lentiviral vectors expressing GFP, progranulin (PGRN) or lysosome‐targeted progranulin (L‐PGRN) under the human PGK promoter. Cultures were maintained for 4 days before fixing and immunostaining. (B) The lenti‐PGK vectors transduced both neurons (MAP2+) and astrocytes (GFAP+), which were the two primary cell types observed in these cultures. (C) Virally expressed PGRN was secreted, while L‐PGRN exhibited no detectable secretion, even in media concentrated 5× using a centrifugal filter. (D, E) Both PGRN (linear mixed effects model, main effect of lentivirus, *F*_(3,556)_ = 3.2966, *p* = 0.02024, PGRN versus GFP, *p* = 0.0404) and L‐PGRN (linear mixed effects model, *p* = 0.0119) stimulated dendritic outgrowth compared to GFP, while untransduced neurons exhibited comparable growth to those transduced with lenti‐GFP (linear mixed effects model, *p* = 0.9745). *n* = 124–159 neurons per group from 4 to 5 independent cultures (one culture did not include untransduced neurons). Violin and box plots represent the distribution of total dendritic length for all neurons analyzed, and dots represent the median length of all neurons from each culture. Scale bars in B and E represent 50 μm.

Using this culture paradigm, we next tested the effects of lentiviral vectors expressing progranulin (PGRN) or L‐PGRN on dendritic outgrowth. These vectors utilized the PGK promoter, which expressed in neurons and astrocytes, the two primary cell types found in our cultures (Figure 2b). Lenti‐PGK‐PGRN increased dendritic outgrowth (Figure 2d), consistent with prior reports on virally expressed progranulin in primary neurons (Gass et al. 2012). Lenti‐PGK‐L‐PGRN also increased dendritic outgrowth, indicating that progranulin secretion is not necessary for this effect and that progranulin's actions in lysosomes promote dendritic outgrowth.

Similar to results with recombinant progranulin, we observed no effect of PGRN or L‐PGRN on dendritic branching or the number of primary dendrites (Figure S1c,d). Thus in further analyses, we focused on increases in total dendritic length, which is a well‐documented effect of progranulin on cultured neurons.

### 3.3 Delivering Progranulin to Neuronal Lysosomes Does Not Promote Dendritic Outgrowth

**FIGURE 3:**
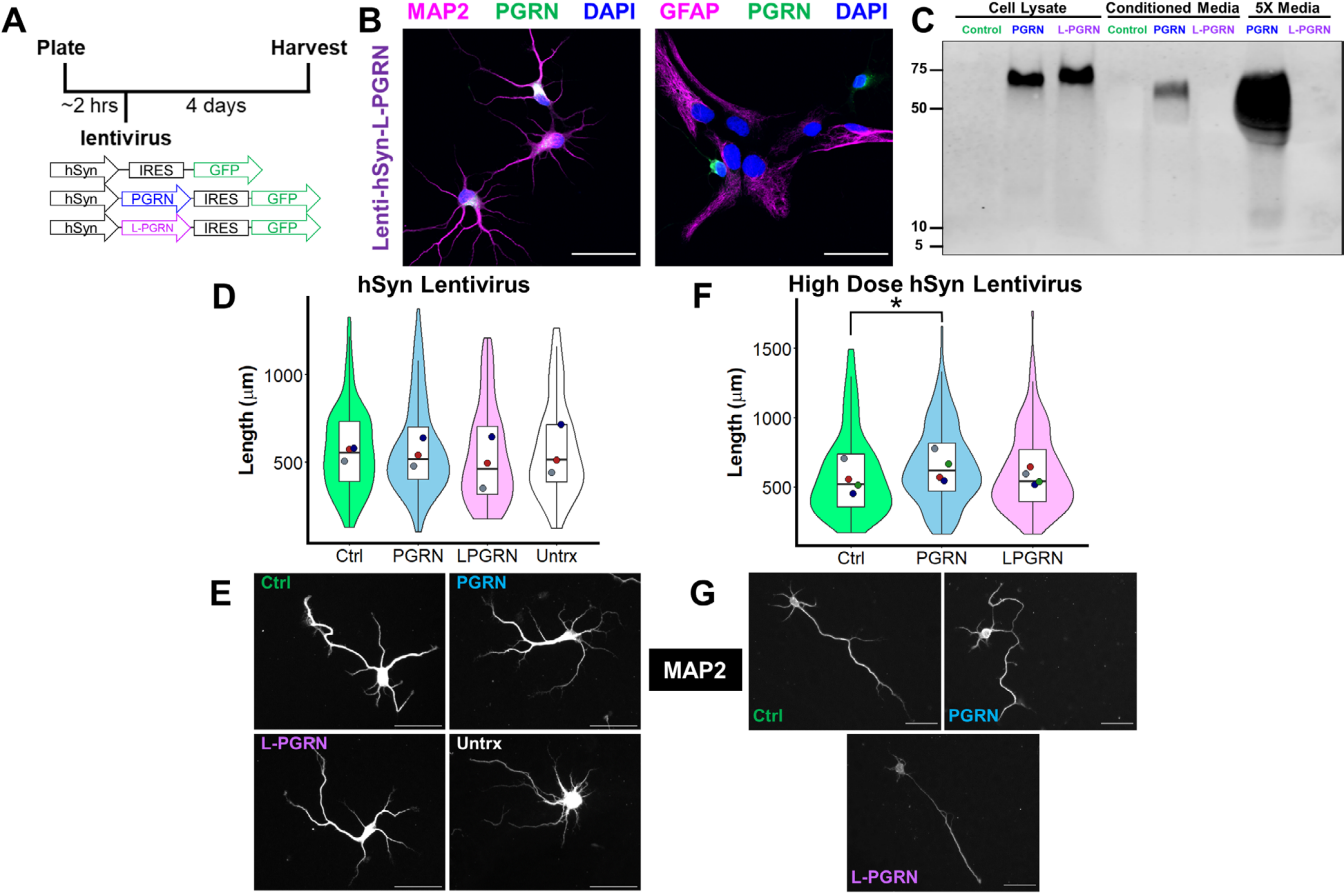
Delivering progranulin to neuronal lysosomes does not stimulate dendritic outgrowth. (A) Primary hippocampal cultures were plated onto coverslips and transduced with hSyn‐IRES‐GFP vectors, with an empty IRES‐GFP vector serving as a control for the PGRN‐IRES‐GFP and L‐PGRN‐IRES‐GFP vectors. Cultures were maintained for 4 days before fixing and immunostaining. (B) The lenti‐hSyn vectors selectively transduced neurons (MAP2+ cells). (C) Virally expressed PGRN was secreted, while L‐PGRN exhibited no detectable secretion, even in media concentrated 5× using a centrifugal filter. (D, E) When administered at a viral dose that gave equivalent levels of PGRN as the lenti‐PGK vector, neither PGRN nor L‐PGRN (linear mixed effects model, main effect of lentivirus, *F*_(3,328)_ = 1.5083, *p* = 0.2122) stimulated dendritic outgrowth compared to GFP. *n* = 76–89 neurons per group from 3 independent cultures. (F, G) When administered at a dose of equivalent viral copies, which produced higher levels of PGRN than the lenti‐PGK vector, PGRN stimulated dendritic outgrowth compared to GFP (linear mixed effects model, main effect of lentivirus, *F*_(2,606)_ = 3.3301, *p* = 0.03644, PGRN vs. Ctrl, *p* = 0.0113), while L‐PGRN did not (linear mixed effects model, *p* = 0.3766). *n* = 202–207 neurons per group from 4 independent cultures. Violin and box plots represent the distribution of total dendritic length for all neurons analyzed, and dots represent median length of all neurons from each culture. Scale bars in B, E, and G represent 50 μm.

Based on our prior work (Davis et al. 2021), we hypothesized that progranulin's stimulation of dendritic outgrowth was mediated by a direct effect on neurons. We tested this hypothesis by transducing cultures with lentiviral vectors using the neuron‐specific hSyn promoter. We initially tested a viral dose of the hSyn vectors at which lenti‐hSyn‐PGRN produced similar levels of progranulin as lenti‐PGK‐PGRN (Figure S2). Neither lenti‐hSyn‐PGRN nor lenti‐L‐PGRN stimulated neuronal outgrowth at this dose (Figure 3d). We then treated cultures using hSyn vectors at the same viral dose as the PGK vectors, which resulted in higher levels of progranulin than produced by the PGK vectors (Figure S1). At this dose, lenti‐hSyn‐PGRN modestly stimulated dendritic outgrowth (Figure 2f), but lenti‐hSyn‐L‐PGRN again had no detectable effect.

In summary, non‐specific delivery of progranulin to lysosomes of primary hippocampal cultures promoted dendritic outgrowth, but specific delivery of progranulin to neuronal lysosomes did not. Furthermore, specific expression of progranulin in neurons only promoted dendritic outgrowth when administered at a dose that produced high levels of progranulin in the culture medium (Figure S2). We thus hypothesized that progranulin may promote dendritic outgrowth through a non‐cell autonomous mechanism.

### 3.4 Delivering Progranulin to Astrocytic Lysosomes Promotes Dendritic Outgrowth

**FIGURE 4:**
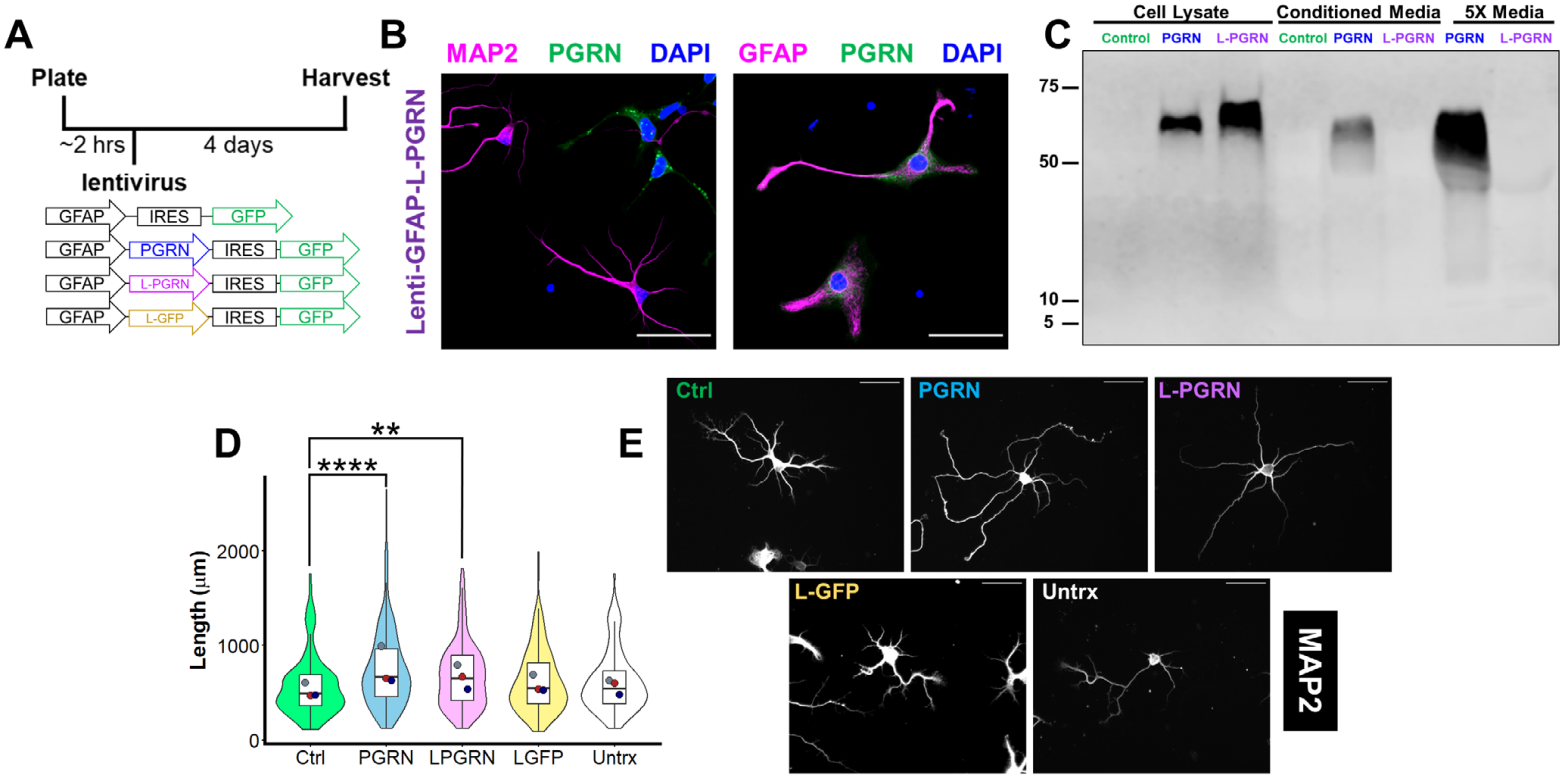
Delivering progranulin to astrocytic lysosomes stimulates dendritic outgrowth. (A) Primary hippocampal cultures were plated onto coverslips and transduced with GFAP‐IRES‐GFP vectors, with the empty vector serving as a control for the PGRN‐IRES‐GFP and L‐PGRN‐IRES‐GFP vectors. Cultures were maintained for 4 days before fixing and immunostaining. (B) Lenti‐GFAP vectors selectively transduced astrocytes (GFAP+ cells). (C) Virally expressed PGRN was secreted, while L‐PGRN exhibited no detectable secretion, even in media concentrated 5× using a centrifugal filter. (D, E) Both PGRN (linear mixed effects model, main effect of lentivirus, *F*_(4,801)_ = 5.4101, *p* = 0.0002662, PGRN vs. Ctrl, *p* < 0.0001) and L‐PGRN (linear mixed effects model, L‐PGRN vs. Ctrl, *p* = 0.00155) stimulated dendritic outgrowth compared to the control vector, while untransduced neurons did not have significantly different growth from those transduced with lenti‐IRES‐GFP (linear mixed effects model, *p* = 0.1539). As an additional control, this experiment included neurons transduced with lysosome‐targeted GFP (L‐GFP), which also did not stimulate growth relative to the control vector (linear mixed effects model, *p* = 0.1443). *n* = 139–193 neurons per group from 3 independent cultures. Violin and box plots represent the distribution of total dendritic length for all neurons analyzed, and dots represent the median length of all neurons from each culture. Scale bars in B and E represent 50 μm.

To test this hypothesis, we generated lentiviral vectors expressing PGRN or L‐PGRN under the GFAP promoter, enabling specific expression of progranulin in astrocytes (Figure 4b). Similar to neurons, astrocytes did not secrete L‐PGRN (Figure 4c). We administered these lenti‐GFAP vectors to our cultures at a dose at which lenti‐GFAP‐PGRN produced similar progranulin levels as lenti‐PGK‐PGRN (Figure S2), and found that both lenti‐GFAP‐PGRN and lenti‐GFAP‐L‐PGRN promoted dendritic outgrowth (Figure 4d). A lysosome‐targeted GFP (L‐GFP) vector failed to reproduce these effects, indicating that L‐PGRN's effects are likely due to lysosomal delivery of progranulin rather than nonspecific effects of delivering excess protein to astrocytic lysosomes (Figure S3e). These results led us to hypothesize that progranulin promotes dendritic outgrowth through actions in astrocytic lysosomes.

### 3.5 Increased Dendritic Outgrowth in Neurons Co‐Cultured With L‐PGRN–Transduced Astrocytes

**FIGURE 5:**
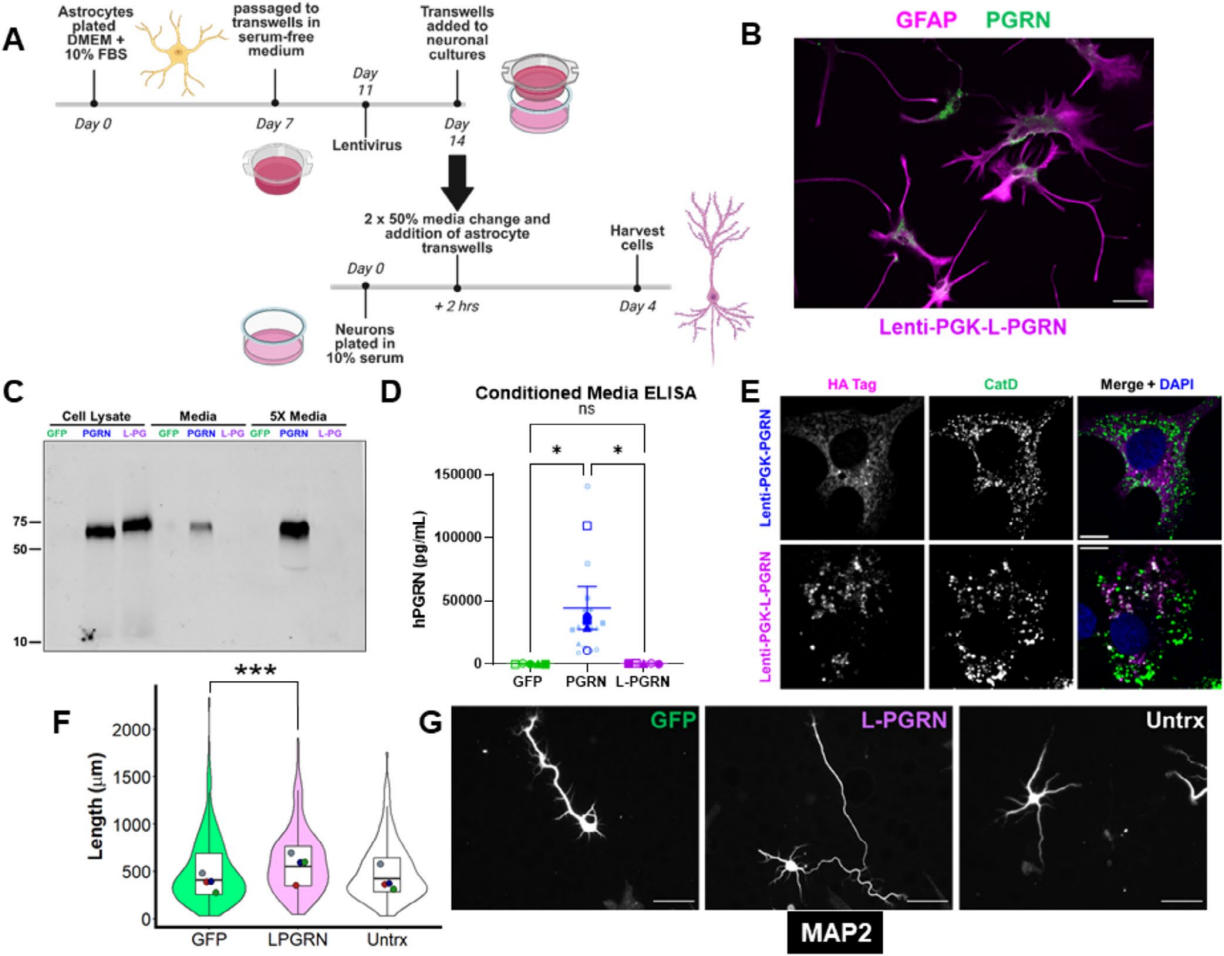
Enhanced dendritic outgrowth in neurons co‐cultured with astrocytes transduced with L‐PGRN. (A) mature hippocampal astrocyte cultures were obtained by plating in DMEM with 10% serum and culturing for 1 week, then passaging to transwell inserts in serum‐free medium and transducing with lenti‐PGK vectors 4 days later. These transwell inserts containing mature astrocytes were placed over new primary hippocampal cultures, which were harvested after 4 days for immunostaining. Representative progranulin immunostaining in astrocytes is shown in (B). (C) PGRN was secreted by astrocytic cultures, while L‐PGRN was not. (D) Lack of progranulin secretion was confirmed by ELISA of conditioned media of several astrocyte cultures. Media from cultures transduced with lenti‐PGK‐GFP exhibited low background signal for human progranulin (hPGRN), which was dramatically increased in cultures transduced with lenti‐PGK‐PGRN (ANOVA main effect of lentivirus, *F*_(2,12)_ = 6.974, *p* = 0.0098, PGRN versus GFP *p* = 0.0123 and PGRN vs. L‐PGRN *p* = 0.0297 by Tukey's post hoc test, *n* = 16 wells from 5 independent cultures). Cultures transduced with lenti‐PGK‐L‐PGRN did not differ from GFP (*p* = 0.877 by Tukey's post hoc test) and also had dramatically less hPGRN in conditioned media than cultures transduced with lenti‐PGK‐PGRN (*p* = 0.0297 by Tukey's post hoc test). (E) L‐PGRN was delivered to lysosomes of primary astrocytes, as shown by colocalization of the HA tag from virally expressed L‐PGRN with the lysosomal protease cathepsin D (CatD). (F, G) Despite the lack of progranulin secretion, neurons co‐cultured with astrocytes transduced with L‐PGRN had greater dendritic outgrowth compared to neurons cultured with GFP‐treated astrocytes (linear mixed effects model, main effect of lentivirus, *F*_(2,580)_ = 7.6264, *p* = 0.0005379, L‐PGRN vs. GFP, *p* = 0.000619) while neurons co‐cultured with untransduced astrocytes had similar dendritic outgrowth as neurons cultured with GFP‐treated astrocytes (linear mixed effects model, *p* = 0.876719). *n* = 180–206 neurons per group from 4 independent cultures. Violin and box plots represent the distribution of total dendritic length for all neurons analyzed, and dots represent median length of all neurons from each culture. Scale bars represent 50 μm in B and G and 10 μm in E. Panel A created at Biorender.com, Arrant (2025) https://BioRender.com/cme038k.

To test this next hypothesis, we adopted a culture strategy that produces mature astrocytes with in vivo‐like transcriptional profiles (Figure 5a) (Wolfes et al. 2017; Wolfes and Dean 2018). Preliminary experiments confirmed that these astrocytes did not secrete L‐PGRN (Figure 5c,d). Furthermore, L‐PGRN was delivered to astrocytic lysosomes (Figure 5e), as we previously observed in neurons (Davis et al. 2021).

We plated astrocytes onto transwell inserts and transduced them with lenti‐PGK‐GFP or lenti‐PGK‐L‐PGRN, with additional astrocytes treated only with PBS (Figure 5a). We then generated new primary hippocampal cultures that were not treated with lentiviral vectors and placed the transwell inserts containing transduced astrocytes over these naïve cultures. After 4 days of co‐culture, neurons cultured with L‐PGRN–transduced astrocytes had significantly more dendritic outgrowth than neurons cultured with GFP‐transduced astrocytes (Figure 5f,g). Neurons cultured with untransduced astrocytes had similar dendritic outgrowth to GFP‐transduced astrocytes. These data support the hypothesis that progranulin acts in astrocytic lysosomes to promote dendritic outgrowth in co‐cultured neurons, and indicate that this effect may be mediated by changes in factors secreted by astrocytes.

### 3.6 L‐PGRN Stimulates Autophagic Flux in Astrocytes

**FIGURE 6:**
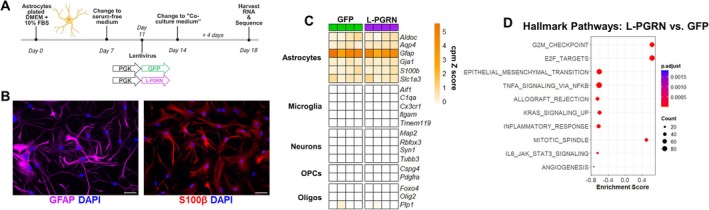
L‐PGRN reduces transcriptomic signatures of cellular reactivity in primary astrocytes. (A) To better understand how delivering progranulin to lysosomes changes the phenotype of primary astrocytes, we cultured mature astrocytes using the same procedure as previously used for neuronal co‐cultures. Instead of co‐culturing with neurons, we switched astrocytes to a “co‐culture medium” identical to that used in co‐cultures. After 4 days in co‐culture medium, we harvested RNA from these astrocytes and conducted bulk RNA sequencing. (B) Nearly all cells in these cultures were immunoreactive for the astrocytic markers GFAP and S100β, and (C) bulk RNA sequencing confirmed the enrichment of astrocyte marker genes versus other cell types. (D) Gene set enrichment analysis of sequencing data using Hallmark pathways suggested potential increases in proliferation and decreases in cellular reactivity. *n* = 4 samples per treatment from 4 independent cultures. Panel A created at Biorender.com, Arrant (2025) https://BioRender.com/560o6wr. Raw counts of genes in panel C are provided in Table S1, and a full list of enriched Hallmark pathways from panel D is provided in Table S2.

To determine how L‐PGRN affects astrocytes, we analyzed mature astrocytic cultures prepared as described for the neuronal co‐culture experiment (Figure 6a). Rather than co‐culturing with neurons, we mimicked these conditions by changing astrocytes to “co‐culture medium”, which was the same formulation used for primary hippocampal cultures, including 2.5% FBS to mimic the serum content after post‐plating media changes. Nearly all cells in these cultures were immunoreactive for astrocytic markers such as GFAP or S100β (Figure 6b).

To investigate how progranulin overexpression affects autophagy‐lysosomal function in astrocytes, we treated astrocytes with lenti‐PGK‐GFP, ‐PGRN, or –L‐PGRN as described above. Four days after transduction, we incubated the transduced astrocytes with vehicle or 50 μM chloroquine for 4 h, then immunoblotted for LC3‐II (Figure S3b,c). Similar to our findings in primary cortical neurons (Davis et al. 2021), L‐PGRN increased LC3‐II levels in chloroquine‐treated astrocytes, indicating an increase in autophagic flux.

We also analyzed the activity of the lysosomal lipid hydrolase β‐hexosaminidase (Hex) in astrocyte lysates and media to investigate changes in general lysosomal activity (lysates) and potential changes to lysosomal exocytosis (media). Similar to prior findings in mouse brain (Arrant et al. 2018, 2019) and in primary neurons (Davis et al. 2021), we found that both PGRN and L‐PGRN overexpression modestly reduced Hex activity in lysates (Figure S3d). However, we observed no change to Hex activity in conditioned media, indicating a lack of robust change to lysosomal exocytosis.

### 3.7 Transcriptional Analysis of L‐PGRN–Transduced Astrocytes

To gain further insight into how delivering progranulin to astrocytic lysosomes changes astrocytes, we conducted RNA sequencing on mature astrocytes prepared as described above (Figure 6a). RNA sequencing of four independent cultures revealed much higher counts of astrocytic markers versus markers of other cell types (Figure 6c), but failed to detect differentially expressed genes between cultures transduced with lenti‐PGK‐GFP or lenti‐PGK‐L‐PGRN after correction for false discovery. To gain insight into the effects of L‐PGRN, we therefore conducted gene set enrichment analysis (GSEA) (Subramanian et al. 2005) using hallmark pathways from the molecular signatures database (Liberzon et al. 2015). The most significant terms from this analysis suggested a potential increase in proliferation and a potential decrease in cellular reactivity in astrocytes treated with L‐PGRN (Figure 6d). A full list of significant Hallmark pathways detected by GSEA is provided in Table S2.

### 3.8 L‐PGRN Does Not Increase Astrocyte Proliferation

To determine whether transduction with PGRN or L‐PGRN increased astrocyte proliferation, we cultured mature astrocytes in serum‐free media, then switched to co‐culture medium as described above. After 3 days in co‐culture medium, we treated cultures with 10 μM BrDU and fixed for immunostaining 24 h later. We observed no change in the proportion of astrocytes immunoreactive for BrDU, though we observed a statistically non‐significant trend for an increase in total cell count with L‐PGRN treatment (Figure S4). Thus, L‐PGRN may have modestly increased astrocyte proliferation at an earlier phase of culture, or may have increased astrocyte survival in culture. However, any such effects were not robust enough to produce a significant increase in cell count at the end of the experimental period.

### 3.9 L‐PGRN Reduces Secretion of PAI‐1

**FIGURE 7:**
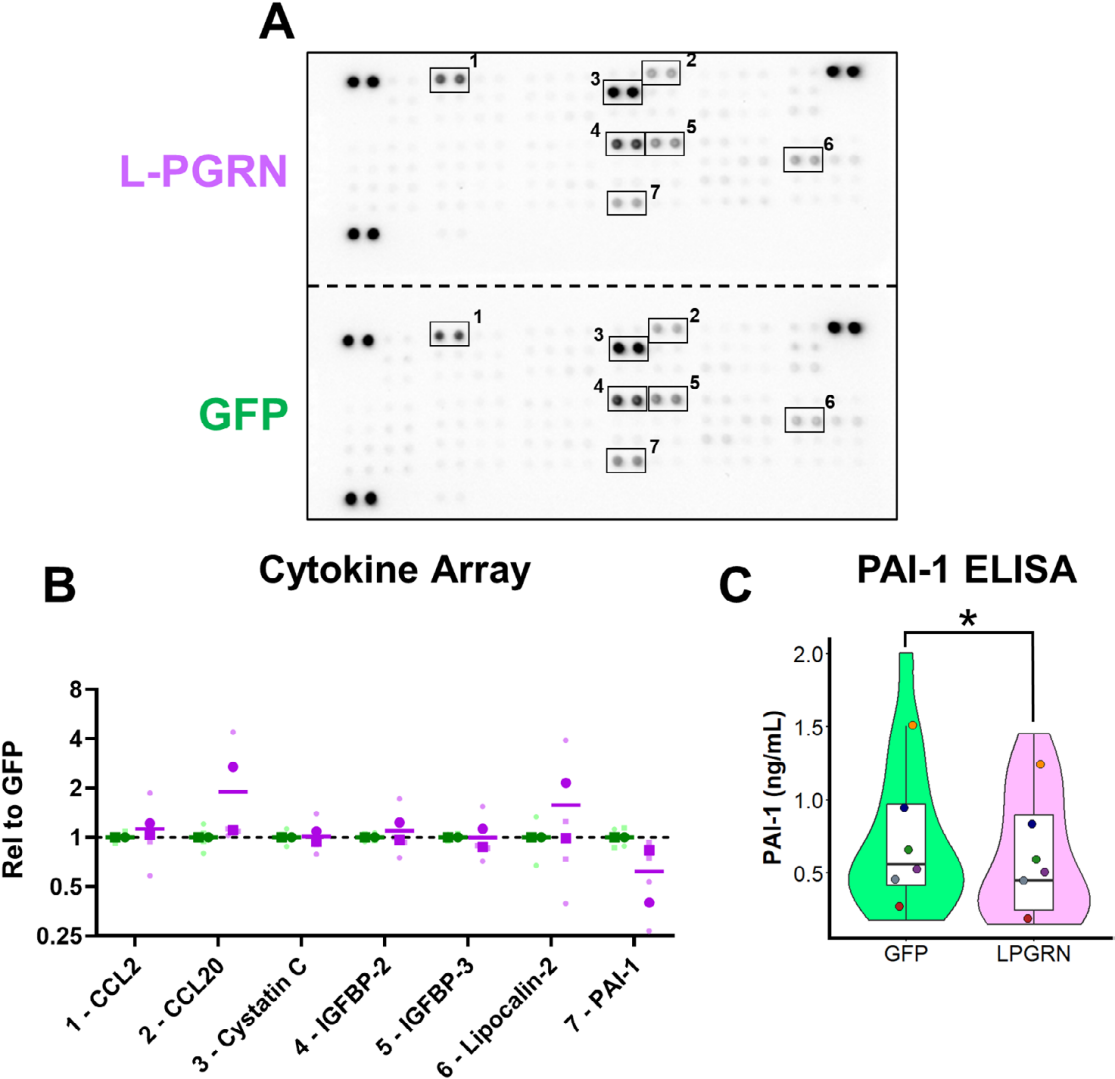
L‐PGRN reduces secretion of PAI‐1 from primary astrocytes. (A) To investigate changes in secreted factors from L‐PGRN–treated primary astrocytes that could enhance neuronal growth, we performed a cytokine array on conditioned media from the transwell experiments described in Figure 5A,F (*n* = 4 samples from 2 independent cultures per lentivirus). (B) Seven factors exhibited robust signal across all blots, and of these only Plasminogen Activator Inhibitor‐1 (PAI‐1) exhibited signs of changes in L‐PGRN cultures versus GFP. The graph in panel B is shown in log2 scale. Large symbols denote culture averages, while small symbols show individual replicates. (C) PAI‐1 ELISA on conditioned media from separate primary astrocyte cultures transduced with lenti‐PGK‐GFP or lenti‐PGK‐L‐PGRN confirmed this effect, with media from L‐PGRN–treated cultures containing less PAI‐1 than cultures transduced with GFP (paired *t*‐test, *t*(5) = 2.785, *p* = 0.0387, *n* = 17–18 samples from six independent cultures) Violin and box plots represent the distribution of all samples, and dots represent the average PAI‐1 levels from each culture.

Based on our findings that L‐PGRN−transduced astrocytes grown on transwells enhance the growth of co‐cultured neurons (Figure 5) and transcriptomic data indicating that L‐PGRN−transduced astrocytes may be less reactive (Figure 6), we hypothesized that L‐PGRN's effects on neuronal growth may be mediated by changes to factors secreted by astrocytes. To screen for any changes to secreted factors, we analyzed conditioned media from the transwell co‐culture experiments (Figure 5) using a rat cytokine array (Figure 7a). We obtained robust signals for 7 proteins on the array, with most exhibiting no clear trends for changes between GFP‐ and L‐PGRN−transduced astrocytes. However, we observed a trend for the reduction of plasminogen activator inhibitor 1 (PAI‐1, also known as Serpin E1). Interestingly, the reduction of PAI‐1 secretion from astrocytes treated with valproic acid has been shown to increase the growth of primary neurons (Cho et al. 2013).

We therefore sought to confirm this result in new cultures. We conducted PAI‐1 ELISA on conditioned media from mature astrocytes cultured with “co‐culture medium” as described for the RNA sequencing study. This analysis confirmed a modest reduction in PAI‐1 levels with L‐PGRN transduction (Figure 7c).

### 3.10 Reducing Astrocyte Numbers Occludes the Effects of L‐PGRN

Taken together, our findings indicate that rather than inducing a neurotrophic phenotype in astrocytes, L‐PGRN may instead reduce secretion of factors that inhibit neuronal growth. Comparison of untreated cultures from all prior experiments (Figures 1, 2, 3, 4, 5) supported this possibility. Untreated neurons from the transwell experiment, which were in proximity to many more astrocytes than other cultures, had lower dendritic length than most other cultures (Figure S5).

While we identified the reduction of PAI‐1 secretion as a potential mechanism by which L‐PGRN enhances dendritic outgrowth, there may also be important changes to other secreted factors in L‐PGRN−transduced astrocytes. To test the general hypothesis that L‐PGRN enhances neuronal growth by inhibiting astrocytic secretion of growth‐inhibiting factors, we therefore investigated the effects of reducing the numbers of astrocytes in primary hippocampal cultures treated with lenti‐PGK‐GFP or lenti‐PGK‐L‐PGRN.

**FIGURE 8:**
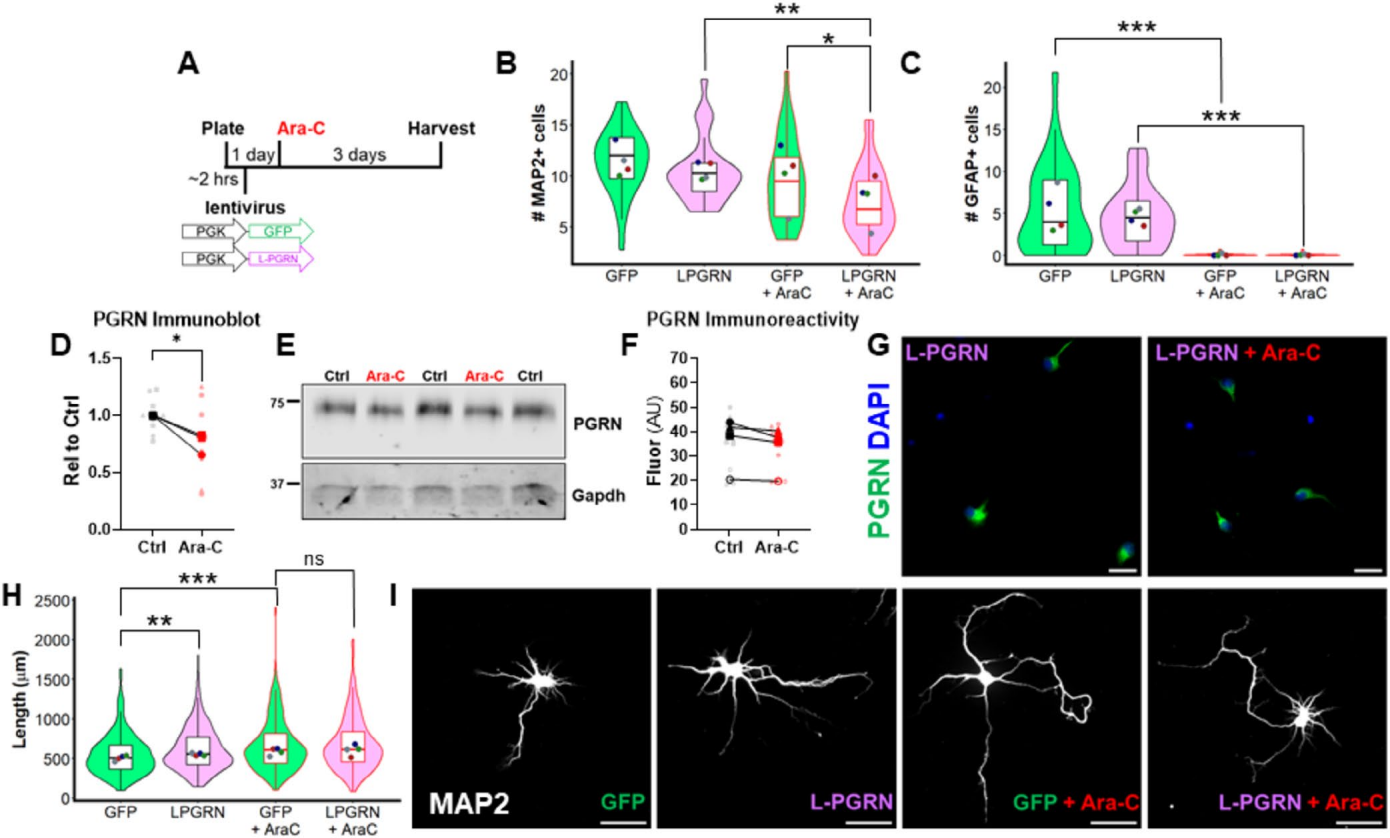
Reducing the number of astrocytes occludes the pro‐growth effects of L‐PGRN. (A) Primary hippocampal cultures were plated onto coverslips, transduced with lenti‐PGK‐GFP or L‐PGRN, then treated with 5 μM Ara‐C approximately 24 h after plating. Cultures were maintained for a total of 4 days. (B) Ara‐C treatment modestly reduced the number of neurons in these cultures (linear mixed effects model main effect of Ara‐C, *F*_(1,108)_ = 16.5707, *p* < 0.0001, ** = *p* = 0.00122 for L‐PGRN vs. L‐PGRN+Ara‐C by Tukey's post hoc test). L‐PGRN–treated cultures treated with Ara‐C also had slightly fewer neurons per visual field than GFP‐treated cultures treated with Ara‐C (linear mixed effects model main effect of lentivirus, *F*_(1,108)_ = 6.3728, *p* = 0.01304, * = *p* = 0.04435 for GFP + Ara‐C vs. L‐PGRN+Ara‐C by Tukey's post hoc test), though there was not a significant difference between lentiviral groups under control conditions (*p* = 0.65663 by Tukey's post hoc test). (C) Ara‐C nearly completely eliminated astrocytes from these cultures (linear mixed effects model main effect of Ara‐C, *F*_(1,108)_ = 74.08, *p* < 0.0001, *** = *p* < 0.001 for GFP vs. GFP + Ara‐C and L‐PGRN vs. L‐PGRN+Ara‐C by Tukey's post hoc test). There was not a significant difference between lentiviral treatment groups in the number of astrocytes per visual field (linear mixed effects model main effect of lentivirus, *F*_(1,108)_ = 0.707, *p* = 0.4023). *n* = 28–29 coverslips from four independent cultures. Violin and box plots represent the distribution of cell count for all coverslips analyzed, and dots represent median cell count from each culture. (D, E) L‐PGRN+Ara‐C wells expressed less progranulin than L‐PGRN + vehicle wells by immunoblot (paired *t*‐test, *t*_(2)_ = 4.403, *p* = 0.0479, *n* = 10–11 replicates from 3 independent cultures). (F, G) However, analysis of progranulin immunoreactivity in neurons revealed a non‐significant trend for lower progranulin (paired *t* test, *t*_(3)_ = 2.237, *p* = 0.1113, *n* = 12 replicates from 4 independent cultures). Scale bars in G represent 100 μm. (H) Analysis of dendritic length in vehicle‐treated cultures replicated our prior finding that L‐PGRN promotes dendritic outgrowth (linear mixed effects model, main effect of lentivirus, *F*_(1,996)_ = 5.1756, *p* = 0.02312, ** = *p* = 0.00802 for GFP vs. L‐PGRN by Tukey's post hoc test), but also revealed that Ara‐C treatment stimulated dendritic outgrowth (linear mixed effects model main effect of Ara‐C, *F*_(1,996)_ = 15.5234, *p* < 0.0001, *** = *p* < 0.001 for GFP vs. GFP + Ara‐C by Tukey's post hoc test) and that among Ara‐C treated neurons, those transduced with L‐PGRN did not have significantly different dendritic growth from those transduced with GFP (linear mixed effects model, *p* = 0.99088). *n* = 215–275 neurons per group from 4 independent cultures. Violin and box plots represent the distribution of total dendritic length for all neurons analyzed, and dots represent median length of all neurons from each culture. Representative images of MAP2 immunostaining are shown in (I) with 50 μm scale bars.

To reduce the number of astrocytes, we treated cultures with 5 μM Ara‐C approximately 24 h after plating (Figure 8a). Imaging of cell‐type–specific markers revealed that Ara‐C treatment modestly reduced the number of neurons (MAP2+ cells) (Figure 8b), but almost completely eliminated astrocytes (GFAP+ cells) from the culture (Figure 8c). Analysis of progranulin levels in L‐PGRN–transduced cultures by immunoblot revealed a modest decrease in progranulin expression with Ara‐C treatment (Figure 8d,e). To determine if this might be related to the change in culture composition or by a true suppression of lentiviral expression, we immunostained cultures for progranulin and compared progranulin immunoreactivity in control and Ara‐C–treated cultures. This analysis revealed a statistically non‐significant trend for reduction in progranulin immunoreactivity (Figure 8f,g), leading us to conclude that Ara‐C treatment does not dramatically suppress expression of L‐PGRN in transduced neurons.

We then analyzed dendritic length in cultures treated with lenti‐PGK‐GFP or L‐PGRN, with and without addition of Ara‐C at DIV1. This analysis replicated our prior finding that lenti‐PGK‐L‐PGRN increased dendritic outgrowth under control conditions (Figure 8h,i). However, neurons from Ara‐C–treated cultures had greater dendritic outgrowth than those from control cultures. Within Ara‐C–treated cultures, L‐PGRN no longer increased dendritic outgrowth compared to GFP. These data suggest that under these culture conditions, astrocytes may suppress dendritic outgrowth, and that L‐PGRN reduces this suppressive effect.

## 4 Discussion

In this study, we found that progranulin stimulates dendritic outgrowth in primary hippocampal neurons through a non‐cell−autonomous mechanism. Our data indicate that progranulin's actions in astrocytic lysosomes reduce the secretion of factors such as PAI‐1 (Cho et al. 2013) that suppress dendritic outgrowth. These data indicate distinct mechanisms underlying progranulin's stimulation of dendritic outgrowth and its neuroprotective effects, which we have shown are mediated by direct actions in neuronal lysosomes in primary cortical neurons (Davis et al. 2021).

An important caveat to this study is that astrocytic phenotypes are dependent on culture conditions, with the presence of serum resulting in phenotypes that differ from those of astrocytes in vivo (Foo et al. 2011). Prior studies have reported that progranulin stimulates the growth of dendrites and axons of primary cortical, hippocampal, and motor neurons incubated with or without serum (Gass et al. 2012; Hyung et al. 2019; Van Damme et al. 2008), showing the robustness of this effect across culture conditions. However, the mechanism by which progranulin stimulates dendritic outgrowth might differ by culture conditions. It is also important to consider that while lysosomes may be progranulin's ultimate site of action in regulating neuronal outgrowth, progranulin secretion may be important for regulating neuron growth in vivo by selectively delivering progranulin to certain neurons or nearby glia (Thomasen et al. 2023; Uesaka et al. 2018).

Despite the potential influence of culture conditions and the limitations of primary culture for modeling the intact brain, our findings are consistent with accumulating data suggesting that progranulin has important effects on astrocytes. Progranulin‐deficient astrocytes are less effective at supporting neuronal maturation (Lee et al. 2023) and have deleterious effects on neurons such as inducing mislocalization of TDP‐43 (de Majo et al. 2023; Marsan et al. 2023). Though astrocytes express low levels of progranulin in vivo (Kaplelach et al. 2023; Petkau et al. 2010), astrocytes become reactive and may lose homeostatic function in degenerated brain regions of patients with FTD due to *GRN* mutations (Gerrits et al. 2022; Marsan et al. 2023). *Grn* ^*−/−*^ mice also develop astrogliosis with age (Ahmed et al. 2010; Filiano et al. 2013; Ghoshal et al. 2012; Marsan et al. 2023). It is currently unclear if these changes are due to loss of progranulin from astrocytes, or if they are induced by microglia, which highly express progranulin (Kaplelach et al. 2023; Petkau et al. 2010) and become reactive in people with *GRN* mutations and *Grn* ^*−/−*^ mice (Ahmed et al. 2010; Filiano et al. 2013; Ghoshal et al. 2012; Lui et al. 2016; Martens et al. 2012; Zhang et al. 2020). It will thus be important for future studies to investigate the mechanism by which progranulin maintains astrocyte homeostasis in the brain.

These findings may also inform mechanisms by which progranulin promotes neuronal outgrowth in the brain. Progranulin‐insufficient mice develop dendritic abnormalities as they age that are consistent with the loss of progranulin's neurotrophic effects. *Grn* ^*−/−*^ mice exhibit reduced spine density in CA1 of the hippocampus, though this is not observed on all genetic backgrounds (Filiano et al. 2013; Petkau et al. 2016, 2012). *Grn* ^*−/−*^ mice also lose thalamic synapses and neurons as they age, which may be driven by reactive microglia (Lui et al. 2016; Zhang et al. 2020) and astrocytes (Marsan et al. 2023). However, *Grn* ^*+/−*^ mice exhibit impaired dendritic arborization and abnormal dendritic spine morphology in the medial prefrontal cortex, despite the absence of robust changes to microglial or astrocytic phenotypes (Arrant et al. 2016; Cook et al. 2024). It will be interesting for future studies to determine if the dendritic abnormalities of *Grn* ^*+/−*^ mice may be mediated by subtle changes to glial cells.

Though we did not assess axonal growth in this study, progranulin insufficiency has been associated with impaired axonal growth in the brain and spinal cord of zebrafish and mice. Progranulin knockdown or knockout in zebrafish reduces axonal growth of motor neurons in the spinal cord of developing zebrafish (Chitramuthu et al. 2010; Laird et al. 2010; Zhu et al. 2021). While *Grn* ^*−/−*^ mice are not reported to exhibit similar developmental impairment of axon growth, they do exhibit impaired regrowth of motor and sensory axons after injury (Altmann, Hardt, et al. 2016; Altmann, Vasic, et al. 2016; Beel et al. 2017; Thomasen et al. 2023). These axonal growth deficits can be mimicked by selective knockout of neuronal progranulin (Beel et al. 2017), and overexpression of neuronal progranulin improves axonal regrowth (Altmann, Hardt, et al. 2016; Altmann, Vasic, et al. 2016), suggesting a key role for neuronal progranulin expression in promoting axonal regrowth after injury. However, due to progranulin's constitutive secretion and uptake, astrocytes or other cell types may take up progranulin secreted from neurons, resulting in changes that promote neuronal growth. Perhaps consistent with this possibility, *Grn* ^*−/−*^ mice exhibit greater astrocyte reactivity after traumatic brain injury, which can be normalized by restoration of progranulin (Menzel et al. 2017; Tanaka et al. 2013; Zheng et al. 2022). However, administration of progranulin to wild‐type mice does not appear to consistently reduce damage from traumatic brain injury (Hummel et al. 2021). Thus, further experiments will be needed to investigate the mechanism by which progranulin promotes axonal regrowth after injury.

In conclusion, this study provides insight into the mechanisms by which progranulin promotes neuronal growth. Our findings indicate that delivering progranulin to astrocytic lysosomes reduces secretion of PAI‐1 and possibly other factors that restrain neuronal growth. Given the influence of culture conditions on astrocytic phenotypes, it will be important for future studies to investigate progranulin's modulation of astrocytes in vivo in the context of aging, injury, and neurodegenerative disease.

## Author Contributions

**Azariah K. Kaplelach:** investigation, formal analysis, writing – review and editing. **Justin A. Hall:** investigation, formal analysis, writing – review and editing. **Wren O. Nader:** investigation, writing – review and editing. **Amelia G. Davidson:** investigation, writing – review and editing. **Margaret D. Ireland:** investigation, writing – review and editing. **Lara Ianov:** formal analysis, writing – review and editing. **Andrew E. Arrant:** conceptualization, investigation, formal analysis, writing – original draft, writing – review and editing.

## Conflicts of Interest

The authors declare no conflicts of interest.

## Peer Review

The peer review history for this article is available at https://www.webofscience.com/api/gateway/wos/peer‐review/10.1111/jnc.70284.

## Supporting information


**Figure S1:** jnc70284‐sup‐0001‐FigureS1.pdf.

**Table S1:** Counts of cell type markers from primary astrocyte cultures.
**Table S2:** Significant Hallmark pathways from RNA sequencing of primary astrocytes transduced with GFP or L‐PGRN.

## Data Availability

Data from bulk RNA sequencing of primary astrocytes has been deposited on the NCBI Gene Expression Omnibus with accession number GSE302849 and are available at https://www.ncbi.nlm.nih.gov/geo/query/acc.cgi?acc=GSE302849. Other data are available from the corresponding author upon request.
