# Supplementary material for: Delivering Progranulin to Astrocytic Lysosomes Promotes Growth of Co‐Cultured Neurons
Source: J Neurochem. 2025 Nov 3;169(11):e70284. doi: 10.1111/jnc.70284 (PMC12580966; doi:10.1111/jnc.70284)
Supplement: Supplementary file 1 — Figure S1: jnc70284‐sup‐0001‐FigureS1.pdf. [file JNC-169-0-s002.pdf]

Supplementary Material for

Delivering progranulin to astrocytic lysosomes promotes growth of co-cultured neurons

Azariah K. Kaplelach, Justin A. Hall, Wren O. Nader, Amelia G. Davidson, Margaret D. Ireland, Lara Ianov, and Andrew E. Arrant

Figures S1–S11

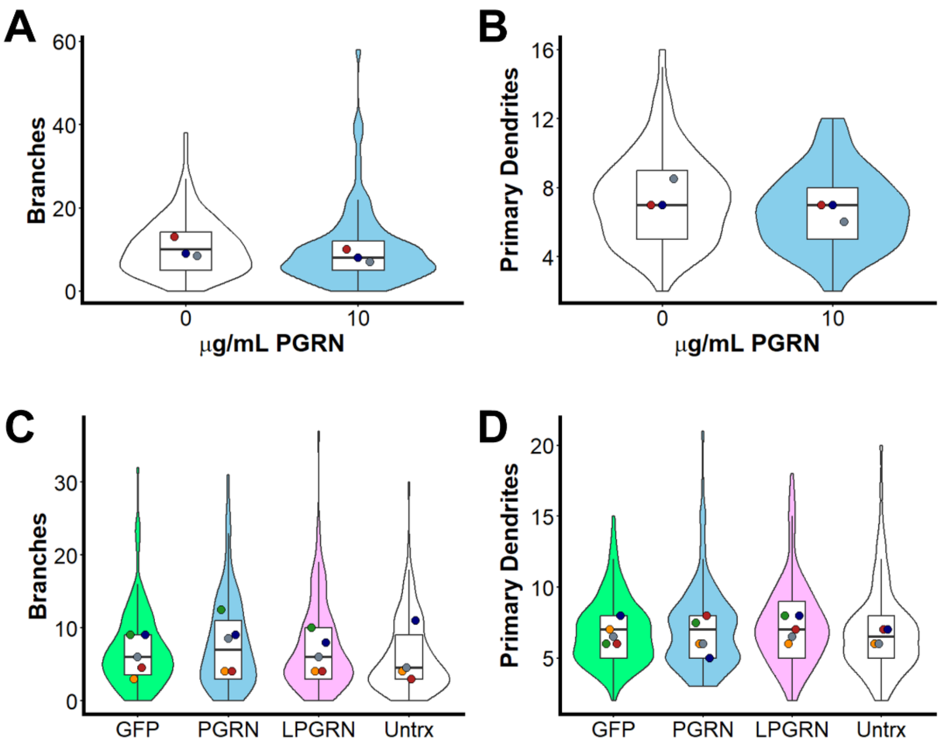

Figure S1 – Recombinant progranulin and lenti-PGK-PGRN do not affect the number of dendritic branches or primary dendrites. A,B, Analysis of neurons treated with recombinant progranulin as shown in Fig. 1 revealed no significant change in the number of dendritic branch points per neuron (linear mixed effects model, $t_{(215)}$ = -1.446, *p* = 0.1495, n = 105 –116 neurons per group from three independent cultures) or the number of primary dendrites per neuron (linear mixed effects model, $t_{(217)}$ = -1.869, *p* = 0.063). **C,D**, Similarly, neither lenti-PGK-PGRN nor lenti-PGK-L-PGRN altered the number of dendritic branches (linear mixed effects model effect of lentivirus, $F_{(3,534)}$ = 1.1849, *p* = 0.3148, n = 124–159 neurons per group from 4–5 independent cultures) or primary dendrites (linear mixed effects model effect of lentivirus, $F_{(3,556)}$ = 0.9016, *p* = 0.4401) in the experiment shown in Fig. 2.

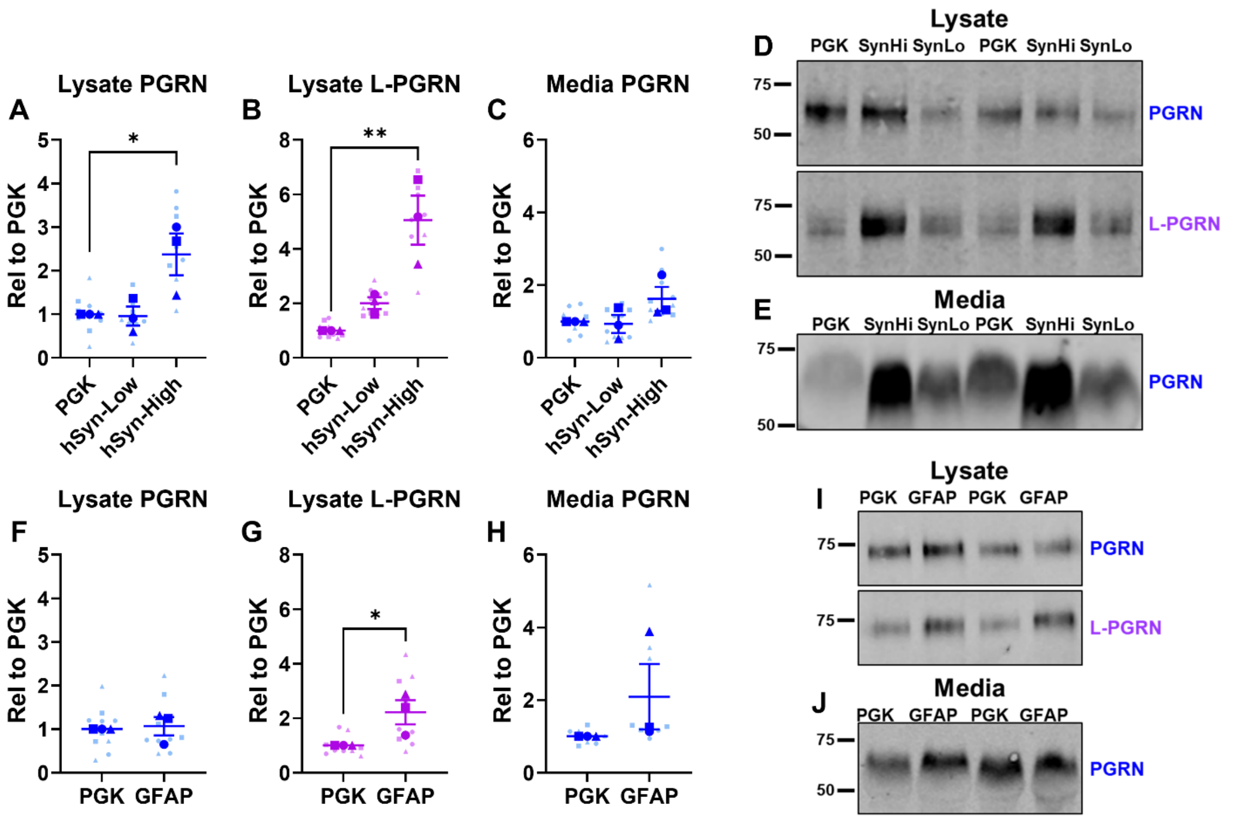

Figure S2 – Comparison of progranulin expression by lentiviral vectors. A, Immunoblotting revealed that a lower viral dose of hSyn-PGRN-IRES-GFP (MOI 1000) produced similar progranulin levels as the dose of PGK-PGRN (MOI 5000) that stimulated dendritic outgrowth. However, an equivalent viral dose of the hSyn-PGRN vector (MOI 5000) resulted in higher progranulin expression (nested ANOVA effect of vector, $F_{(2,6)} = 8.618$, $p = 0.0172$, * = p = 0.0203 by Dunnett's post-hoc test). **B**, hSyn-L-PGRN also produced higher progranulin levels than PGK-L-PGRN at MOI 5000, with a similar trend at MOI 1000 (nested ANOVA effect of vector, $F_{(2,6)} = 16.18$, $p = 0.0038$, ** = p = 0.0028 by Dunnett's post-hoc test). **C**, No L-PGRN was detected in media from any vector, but the hSyn PGRN vectors produced a similar trend for higher media PGRN at MOI 5000 ($F_{(2,6)} = 2.609$, nested ANOVA effect of vector, $p = 0.153$). For hSyn samples, n = 8–10 replicates from 3 independent cultures. **F**, Similarly, a lower viral dose of GFAP-PGRN (MOI 1000) produced similar progranulin levels as PGK-PGRN (MOI 5000) (nested *t* test, $t_{(4)} = 0.4163$, $p = 0.8907$), **G**, while GFAP-L-PGRN (MOI 1000) produced higher levels of progranulin than PGK-L-PGRN (nested *t* test, $t_{(4)} = 3.091$, $p = 0.0366$). **H**, No L-PGRN was detected in media from either vector, but GFAP-PGRN at MOI 1000 produced a non-significant trend for higher progranulin in culture media than PGK-PGRN (nested *t* test, $t_{(4)} = 1.216$, $p = 0.2909$), with only one of three cultures showing signs of increased PGRN. For GFAP samples, n = 9–10 replicates from 3 independent cultures.

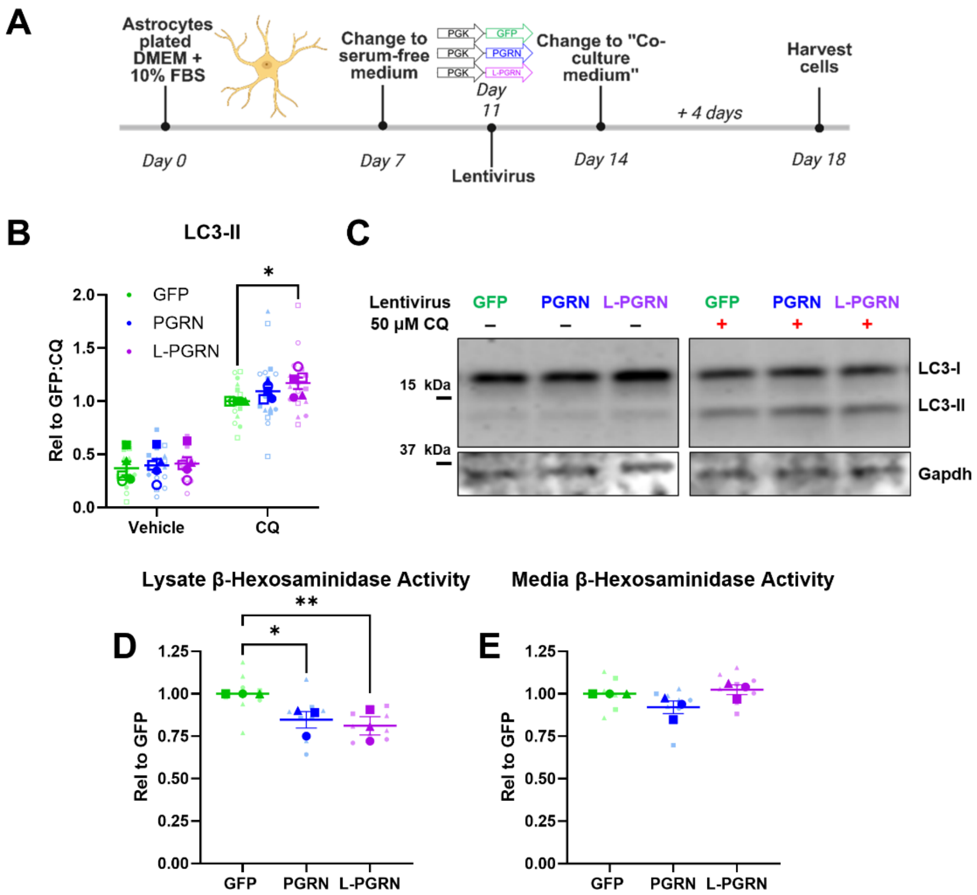

Figure S3 – Effects of L-PGRN on autophagic flux and lysosomal enzyme activity. A, Primary astrocytes were cultured, transduced with lentivirus, and then changed to media mimicking the transwell culture conditions shown in Fig. 5. **B,C**, To assess autophagic flux, astrocytes were incubated for four hours in vehicle or 50 μM chloroquine, then harvested for immunoblot. Astrocytes transduced with L-PGRN exhibited higher LC3-II immunoreactivity than astrocytes transduced with GFP, which is consistent with increased autophagic flux (Nested ANOVA, $F_{(5,24)} = 55.0$, $p < 0.0001$, * = $p = 0.0335$ by Sidak's post-hoc test, n = 17 samples per group from 5 independent cultures). **D**, To analyze lysosomal enzyme activity, we analyzed β-hexosamindase (Hex) as a representative hydrolase regulated by progranulin. Analysis of astrocytic lysates revealed that transduction with both PGRN and L-PGRN reduced Hex activity (Nested ANOVA, $F_{(2,21)} = 5.984$, $p = 0.0088$, * = $p = 0.042$, ** = $p = 0.0059$ by Dunnett's post-hoc test, n = 8 samples from 3 independent cultures). **E**, However, analysis of conditioned media from these same cultures revealed no significant effect of either PGRN or L-PGRN transduction (Nested ANOVA, $F_{(2,21)} = 2.276$, $p = 0.1274$). Panel **A** created at Biorender.com, Arrant, A. (2025).

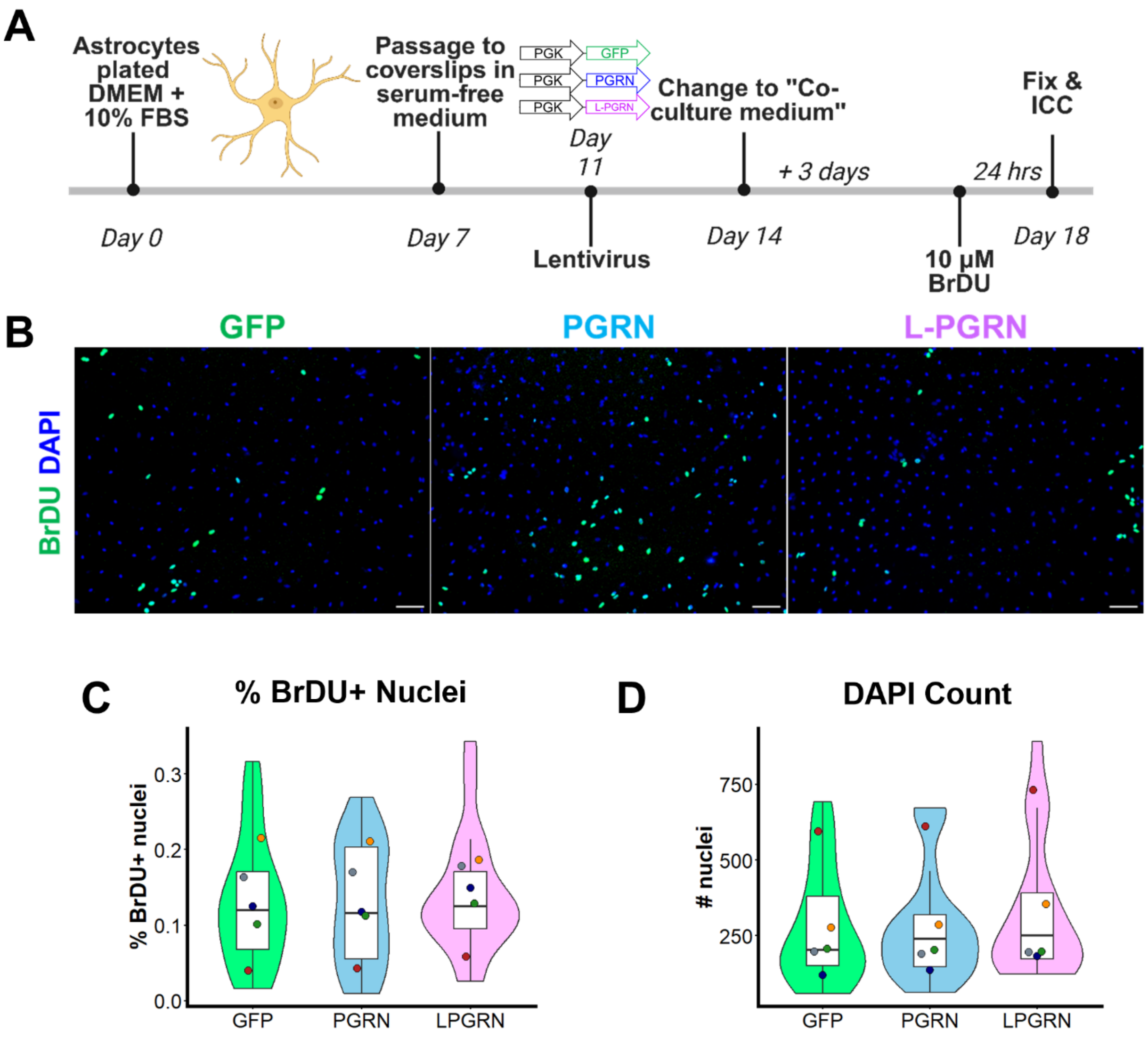

Figure S4 – Delivering progranulin to astrocytic lysosomes does not significantly increase proliferation.

A, Primary astrocytes were cultured and treated with lentiviruses using a similar protocol as for the co-culture and sequencing expeirments. On the final day before harvesting, astrocytes were treated with 10 µM BrDU, then fixed for immunostaining after 24 hours. Representative images of BrDU immunoreactivity are shown in **B** with 100 µm scale bars. **C**, There were no significant change in the percentage of total nuclei that were immunoreactive for BrDU (linear mixed effects model, main effect of lentivirus, $F_{(2, 53)} = 0.728$, $p = 0.4877$). **D**, The total number of nuclei also did not significantly differ between groups (linear mixed effects model, main effect of lentivirus, $F_{(2, 53)} = 1.6871$, $p = 0.1948$). n = 20 coverslips from 5 independent cultures. Panel **A** created at Biorender.com, Arrant, A. (2025).

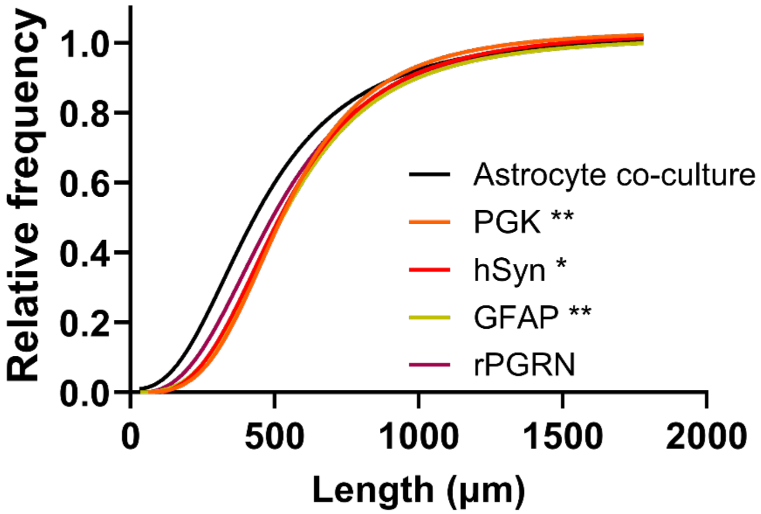

Figure S5 – Comparison of growth across cultures reveals reduced growth of neurons co-cultured with additional astrocytes. Analysis of dendritic growth of untreated neurons from all experiments revealed that neurons co-cultured with astrocytes on transwell inserts exhibited less dendritic outgrowth than neurons from most other experiments. Kolmogorov-Smirnov test *p* values for astrocyte co-culture vs. PGK = 0.0019, vs. hSyn = 0.017, vs. GFAP = 0.0024, and vs. recombinant progranulin (rPGRN) = 0.1913. n = 76–211 neurons from 3–4 independent cultures per experiment.

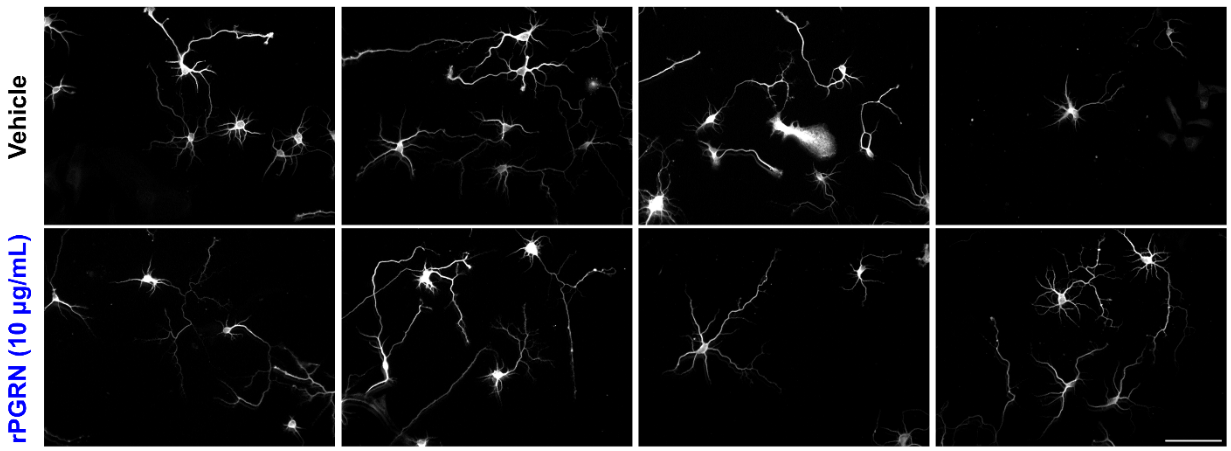

Figure S6 – Additional representative images of neurons from the recombinant progranulin experiment in Figure 1. All images shown at the same scale. Scale bar = 100 µm.

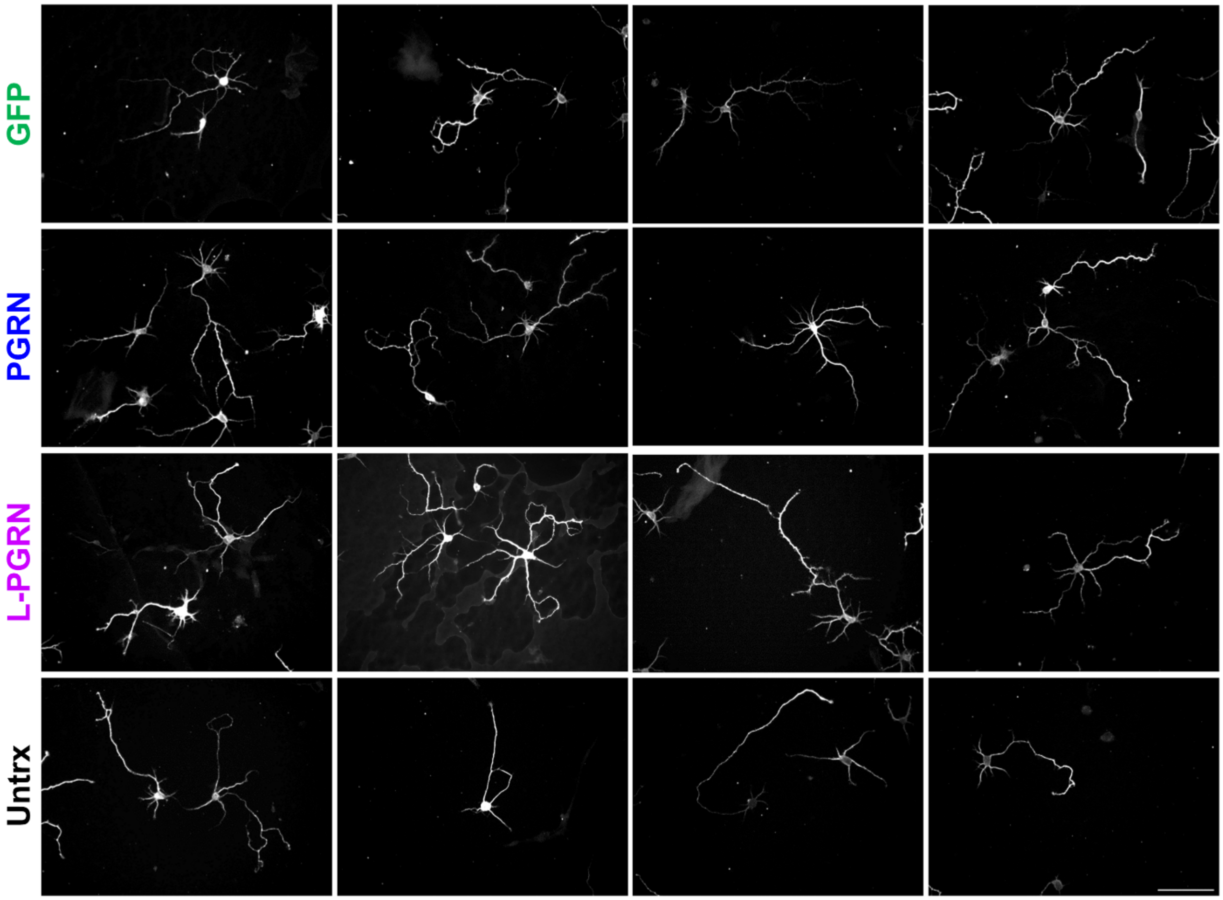

Figure S7 – Additional representative images of neurons from the lenti-PGK experiment in Figure 2. The third PGRN image, the fourth L-PGRN image, and the fourth Untreated image are uncropped versions of images appearing in Fig. 2. All images shown at the same scale. Scale bar = 100 μm.

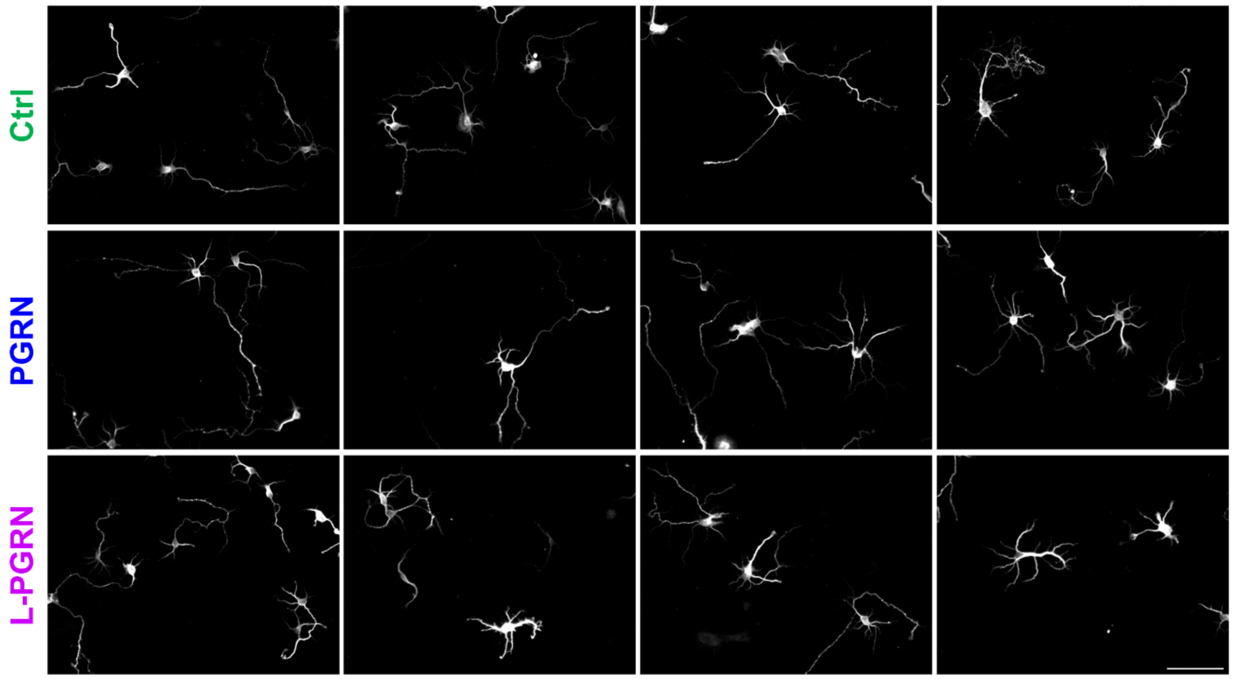

Figure S8 – Additional representative images of neurons from the high-dose lenti-hSyn experiment in Figure 3. All images shown at the same scale. Scale bar = 100 µm.

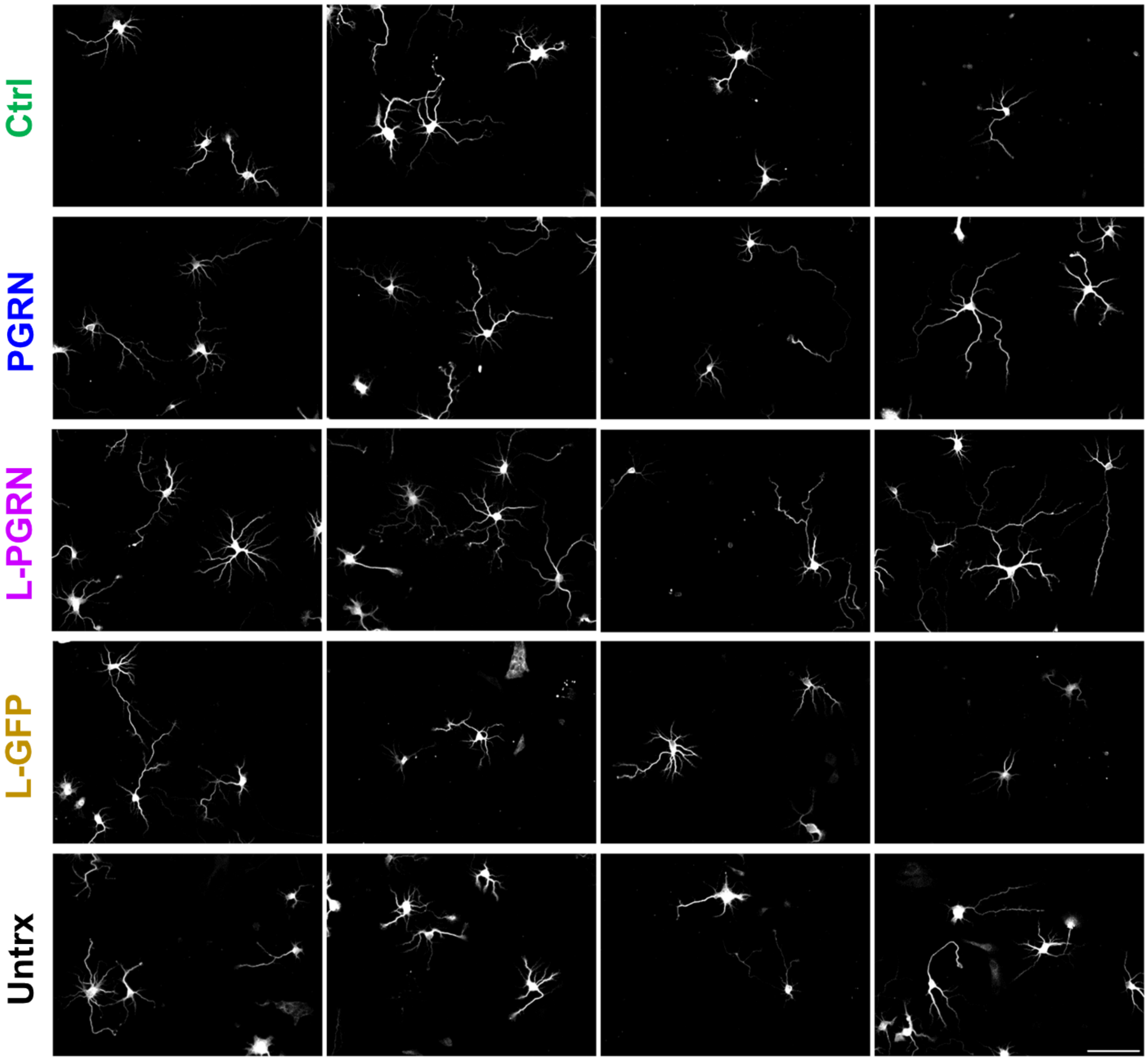

Figure S9 – Additional representative images of neurons from the lenti-GFAP experiment in Figure 4. All images shown at the same scale. Scale bar = 100 μm.

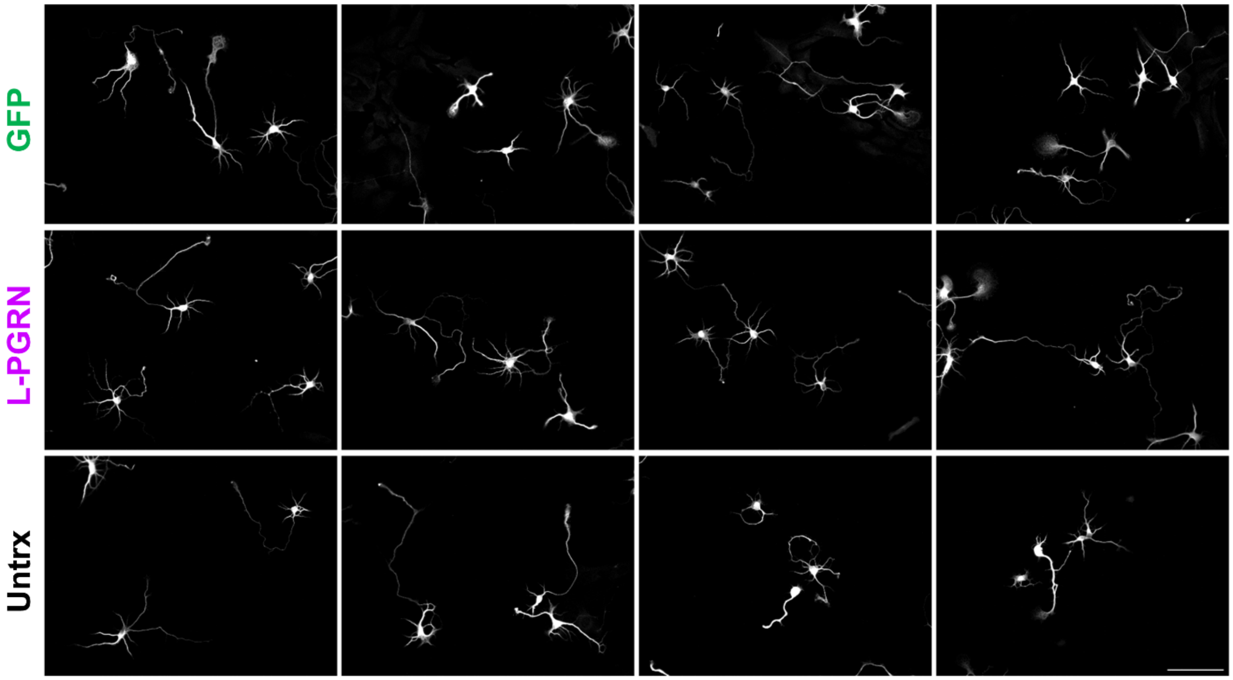

Figure S10 – Additional representative images of neurons from the co-culture experiment in Figure 5. All images shown at the same scale. Scale bar = 100 μm.

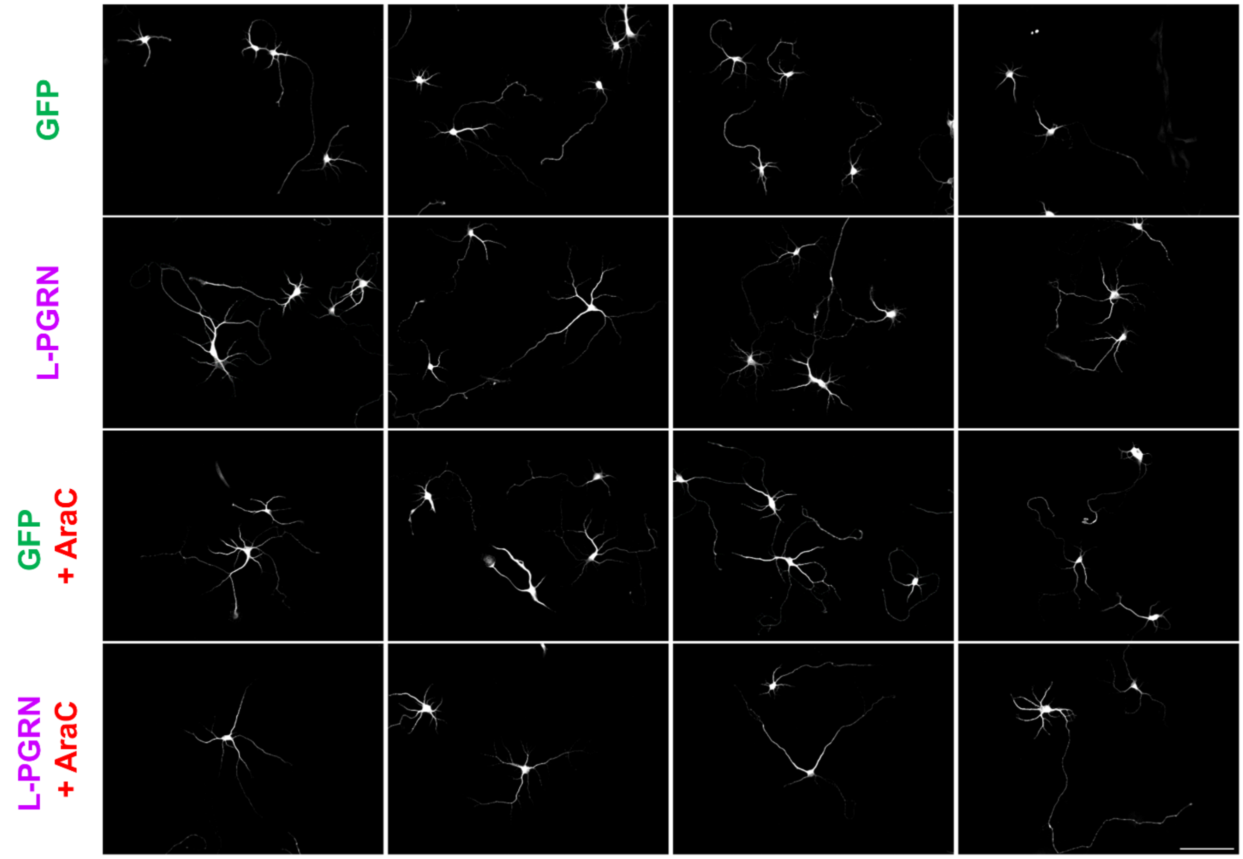

Figure S11 – Additional representative images of neurons from the astrocyte depletion experiment in Figure 8. All images shown at the same scale. Scale bar = 100 µm.
